# Engineering Streptavidin and a Streptavidin-Binding Peptide with Infinite Binding Affinity and Reversible Binding Capability: Purification of a Tagged Recombinant Protein to High Purity via Affinity-Driven Thiol Coupling

**DOI:** 10.1371/journal.pone.0139137

**Published:** 2015-09-25

**Authors:** Dawson Fogen, Sau-Ching Wu, Kenneth Kai-Sing Ng, Sui-Lam Wong

**Affiliations:** Department of Biological Sciences, University of Calgary, Calgary, Alberta, Canada; National Taiwan University, TAIWAN

## Abstract

To extend and improve the utility of the streptavidin-binding peptide tag (SBP-tag) in applications ranging from affinity purification to the reversible immobilization of recombinant proteins, a cysteine residue was introduced to the streptavidin mutein SAVSBPM18 and the SBP-tag to generate SAVSBPM32 and SBP(A18C), respectively. This pair of derivatives is capable of forming a disulfide bond through the newly introduced cysteine residues. SAVSBPM32 binds SBP-tag and biotin with binding affinities (K_d_ ~ 10^-8^M) that are similar to SAVSBPM18. Although SBP(A18C) binds to SAVSBPM32 more weakly than SBP-tag, the binding affinity is sufficient to bring the two binding partners together efficiently before they are locked together via disulfide bond formation–a phenomenon we have named affinity-driven thiol coupling. Under the condition with SBP(A18C) tags in excess, two SBP(A18C) tags can be captured by a tetrameric SAVSBPM32. The stoichiometry of the disulfide-bonded SAVSBPM32-SBP(A18C) complex was determined using a novel two-dimensional electrophoresis method which has general applications for analyzing the composition of disulfide-bonded protein complexes. To illustrate the application of this reversible immobilization technology, optimized conditions were established to use the SAVSBPM32-affinity matrix for the purification of a SBP(A18C)-tagged reporter protein to high purity. Furthermore, we show that the SAVSBPM32-affinity matrix can also be applied to purify a biotinylated protein and a reporter protein tagged with the unmodified SBP-tag. The dual (covalent and non-covalent) binding modes possible in this system offer great flexibility to many different applications which need reversible immobilization capability.

## Introduction

Streptavidin-biotin technology has been widely used in many *in vitro* and *in vivo* applications including the capture and immobilization of biotinylated biomolecules, cell imaging, drug delivery, radioimmunotherapy, the generation of artificial cellulosomes and the building of nanostructures [1–6]. Streptavidin has also been successfully modified to create artificial metalloenzymes [7]. The core technology underlying these many applications is based on the strong non-covalent interaction between biotin and streptavidin, a homotetrameric protein with a biotin binding site in each subunit [8, 9]. This interaction (K_d_ of 4 x10^-14^ M) is one of the strongest non-covalent interactions known [8]. With the development of short peptide streptavidin-binding tags which mimic the binding of biotin to streptavidin without the requirement of biotinylation, the streptavidin technology platform has expanded to include the development of affinity matrices for the purification of tagged recombinant proteins. Several tags with streptavidin-binding affinity in the range from μM (Strep-tag II) to nM [nano-tag and the streptavidin-binding peptide tag (SBP-tag)] are available [10–12]. The SBP-tag is particularly attractive since it has a high binding affinity (K_d_ of 2.5 nM) and can be introduced to recombinant proteins at various positions, including the N- and C-termini as well as many internal locations. SBP-tagged proteins can be affinity purified in one step using wild-type streptavidin agarose matrix [12]. The major drawback of this technology is that the affinity matrix can be used only once, since biotin is typically used as an effective competitor to elute SBP-tagged proteins from the matrix and the tight binding of biotin to streptavidin makes it impractical to remove biotin from the column. With the recent development of SAVSBPM18, an engineered form of streptavidin that can bind both SBP-tag and biotin with affinities in the range of 10^−8^ M [13], we have shown that a SAVSBPM18-based affinity matrix can be used to purify either SBP-tagged or biotinylated biomolecules in a manner where the column can be easily regenerated and reused.

For all of the streptavidin-binding tags, the ability to bind to streptavidin in a reversible manner is a key strength. However, as this reversibility depends on the relatively weak binding affinity to streptavidin, it can also be a key weakness for many applications where a more stable binding interaction is required. None of the peptide tags can bind to streptavidin as strongly as biotin; in fact, biotin binds nearly 10^5^-fold better than the most tightly binding peptide. Therefore, these tags cannot be applied effectively for the purpose of immobilization. It would be ideal to have a designer streptavidin-SBP tag system which allows the engineered SBP-tag to have infinite affinity to streptavidin via covalent bond formation. With this feature, the peptide-tagged biomolecules could be immobilized to streptavidin with a bond strength that is even stronger than the traditional streptavidin-biotin interaction. Under appropriate conditions, breakage of the covalent bond between streptavidin and SBP tag would also be desirable. This would allow reversible interactions between these molecules. Therefore, this system would allow both immobilization and reversible binding to take place under different conditions. In this study, we report the successful development of a designer streptavidin-SBP tag system designated SAVSBPM32-SBP(A18C) with the above-mentioned desirable features. A cysteine residue was introduced to strategic positions in each of SAVSBPM18 and SBP-tag to develop such a system. Application of this designer system for protein purification shows that this system offers several advantages. First, the SAVSBPM32 matrix is backward compatible with the wild-type SBP-tag. Therefore, it can be applied to purify proteins tagged with either SBP, SBP(A18C) or biotin as well as other biotinylated molecules. Second, under optimized wash conditions, the new system does not show protein leakage in the wash fractions even when an affinity column is overloaded with the tagged protein, a problem previously observed in the SAVSBPM18-SBP system [13]. This newly developed system further advances the streptavidin-SBP technology to have the flexibility for both immobilization and reversible binding applications. A novel two-dimensional electrophoresis method was also developed to examine the stoichiometry of the SAVSBPM32-SBP(A18C) complexes. This method can be applied to other systems for analyzing the composition of disulfide-bonded protein complexes.

## Materials and Methods

### Construction of pSSAVSBPM32, pSSAVSBPM32F, pSSAVSBPM96 and pWB980-BLA-L-SBP(A18C)

The genes encoding the SAVSBPM32, SAVSBPM32F and SAVSBPM96 versions of streptavidin were chemically synthesized by Bio Basic Canada Inc. (Ontario, Canada). These genes were individually cloned in *E*. *coli* pUC57 to generate pUC57-SAVSBPM32, pUC57-SAVSBPM32F and pUC57-SAVSBPM96, respectively. Each of these plasmids was digested by *Pst*I and *Bcl*I to release a DNA fragment encoding the streptavidin mutein. Insertion of the SAVSBPM32 fragment to the *Pst*I and *Bcl*I digested pSSAV [14] generated pSSAVSBPM32. Construction of pSSAVSBPM32F and pSSAVSBPM96 was carried out by the same approach. In these streptavidin expression vectors, P43, a strong and constitutively expressed promoter, directs the transcription. The *B*. *subtilis* levansucrase (SacB) signal peptide is applied for secretion. For the construction of pWB980-BLA-L-SBP(A18C), a synthetic gene designated BLA-L-SBP(A18C) was also chemically synthesized by Bio Basic Canada Inc. A 1,060-bp fragment carrying this synthetic gene was released from pUC57-BLA-L-SBP(A18C) by a *Bsa*BI and *Nhe*I double digestion. This fragment was exchanged with the *Bsa*BI/*Nhe*I fragment in pWB980-BLA-L-FLSBP [15] to generate pWB980-BLA-L-SBP(A18C). In this construct, a 19-amino-acid linker with the sequence of IDPAGTSPSTPEGPSTPSN was introduced between TEM-1 β-lactamase and the SBP(A18C) tag.

### *Bacillus subtilis* expression system

*Bacillus subtilis* WB800, the engineered 8-protease deficient strain [16], was used throughout this study as the bacterial expression host for the secretory production of various streptavidin muteins (SAVSBPM18 [13], SAVSBPM32, SAVSBPM32F, SAVSBPM96) and β-lactamase fusions (BLA-L-SBP [15], BLA-L-SBP(A18C), and BLA-L-CPFB(-2) [17]).

### Production and purification of streptavidin muteins

Cells were cultivated for 16 hours at 30°C in the super-rich medium (25 g L^-1^ tryptose, 20 g L^-1^ yeast extract, 3 g L^-1^ dipotassium hydrogen orthophosphate, pH 7.5) [18] containing 10 μg ml^-1^ kanamycin. The culture supernatant containing secreted proteins was concentrated using an Amicon ultra-15 centrifugal filter (10,000 MWCO, Millipore) and dialyzed in physiological buffered saline (PBS, 0.1 M sodium phosphate, 0.15 M sodium chloride, pH 7.5). SAVSBPM18 was affinity purified using biotin-agarose column as previously described [13]. To affinity purify streptavidin muteins carrying cysteine residues (*i*.*e*. SAVSBPM32, SAVSBPM32F and SAVSBPM96), tris(2-carboxyethyl)phosphine (TCEP, Sigma) was added to the concentrated culture supernatant at a final concentration of 2 mM and left for 30 minutes at 23°C. The samples were then loaded onto a biotin-agarose (Sigma, Canada) column. The column was washed with 4 column volumes of PBS containing 2 mM TCEP. Bound proteins were then eluted with 6 column volumes of PBS containing 5 mM D-biotin (Sigma). Fractions containing purified streptavidin muteins were pooled together and concentrated on Amicon ultra-15 centrifugal filters. Excess biotin in the purified samples was then removed by dialysis in PBS. For SAVSBPM32F, 1 mM phenylmethylsulfonyl fluoride (PMSF), a serine protease inhibitor, was added to the concentrated culture supernatant and purified protein sample to limit the proteolytic degradation of the FLAG tag.

### Purification of BLA-L-SBP, BLA-L-SBP(A18C) and BLA-L-CPFB(-2) by ion exchange chromatography

BLA-L-SBP and BLA-L-SBP(A18C) were purified from the culture supernatants using a DEAE-Sepharose Fast Flow column as previously described [15]. BLA-L-CPFB(-2) was purified to homogeneity via a two-step ion-exchange chromatography involving a DEAE column followed by a CM Sepharose column as described previously [17].

### Application of the SAVSBPM32 affinity chromatography for BLA-L-SBP(A18C) purification

Purified SAVSBPM32 was immobilized to the activated Affi-gel 15 media (BioRad, Canada) as described previously [13] and washed with two column volumes of PBS containing 2 mM TCEP followed by a final wash with 2 column volumes of PBS before use. Purification of BLA-L-SBP(A18C) by SAVSBPM32 affinity chromatography was performed under both the overloaded (150% the theoretical binding capacity of the column) and non-overloaded (20% the theoretical binding capacity of the column) conditions with modifications [13]. Briefly, the amount (μg) of BLA-L-SBP(A18C) loaded to the column was based on the theoretical binding capacity of the matrix. This was estimated on the assumption that one BLA-L-SBP(A18C) molecule binds across two subunits in the SAVSBPM32 tetramer. Thus, 1 μg of SAVSBPM32 dimer (Mr = 33,102) will bind 1.061 μg of BLA-L-SBP(A18C) (Mr = 35,113.8). Crude BLA-L-SBP(A18C) was dialyzed in PBS and the concentration was semi-quantified (described below). Samples were then reduced using immobilized TCEP disulfide reducing gel (Pierce) for 1 hour at 23°C with occasional agitation and separated from the immobilized TCEP by centrifuging the mixture at 1,000 g for 1 minute. For the non-overloaded purification condition, an aliquot containing 176 μg of reduced BLA-L-SBP(A18C) was loaded to a 1-ml SAVSBPM32 column. Binding was maximized by capping the column and incubating the mixture for 1 hour at 23°C. Following the incubation, unbound protein was removed by washing the column with 6 column volumes of PBS. Bound protein was then eluted using 6 column volumes of S-Elution buffer (PBS, 2 mM TCEP, 5 mM D-biotin). The first column volume of S-Elution buffer was incubated in the column for 30 minutes prior to elution. The column was regenerated with 10 column volumes of PBS. The same procedure was used for the overloaded condition with the exception that 1,320 μg of reduced BLA-L-SBP(A18C) was loaded to the regenerated SAVSBPM32 column. To optimize the wash conditions, the purification procedure was performed under the overloaded condition with the supplement of one [2% (v/v) Tween-20, 300 mM KCl or 5 mM biotin] or two [300 mM KCl + 5 mM biotin] additional components in PBS. Purification of BLA-L-SBP by immobilized SAVSBPM18 or SAVSBPM32, and purification of BLA-L-SBP(A18C) by immobilized SAVSBPM18 followed a similar approach.

### Application of the SAVSBPM32 affinity chromatography for purification of biotinylated maltose binding protein

Maltose binding protein (MBP) with a C-terminal biotinylation tag (MBP-AviTag fusion, Avidity, USA) was used as a model protein for the purification study. This protein was enzymatically biotinylated with the *E*. *coli* biotin ligase (BirA) [19] as described previously. Biotinylated MBP was purified using Pierce monomeric avidin agarose (Thermo Scientific) and dialyzed in PBS to remove biotin. A crude sample generated by mixing the soluble fraction of *B*. *subtilis* WB800 cell extract with pure biotinylated MBP was loaded onto SAVSBPM32 matrix. The column was washed with 6 column volumes of PBS and the bound protein was eluted by PBS containing 5 mM biotin. The column was regenerated by washing with 10 column volumes of PBS.

### Quantification of proteins

Concentrations of BLA-L-SBP, BLA-L-SBP(A18C) and BLA-L-CPFB(-2) in crude supernatant samples were quantified using standard curves generated on SDS-polyacrylamide gel electrophoresis (SDS-PAGE). Varying amounts of crude supernatant and known concentrations of BSA (New England BioLabs) were analyzed by SDS-PAGE. Following Coomassie blue R250 staining of the gel, the band intensities for each sample were analyzed using ImageJ [20]. Band intensities of the known BSA concentrations were used to generate the standard curve. Quantification of purified proteins was performed using the spectrophotometric method. The absorbance of each sample at 280 nm was measured using a NanoDrop 1000 spectrophotometer (Thermo Scientific). The concentration of each protein was determined using extinction coefficient calculated from the amino acid composition using ProtParam [21, 22].

### Reducing and non-reducing SDS-polyacrylamide gel electrophoresis (SDS-PAGE)

The ability of the studied proteins to form intermolecular disulfide bonds was tested using reducing and non-reducing SDS-PAGE [17]. Briefly, three sets of samples were prepared in PBS. Set 1 contained 1 μM of the tetrameric streptavidin muteins. Set 2 contained 4 μM of SBP, SBP(A18C) or CPFB(-2) tagged BLA and set 3 contained both 1 μM of the tetrameric streptavidin variant and 4 μM of the tagged BLA. These samples were reduced in the presence of 8 mM immobilized TCEP (Pierce, Rockford, IL) for 1 hour at 23°C and then separated from the reductant by centrifugation (1,000 g for 1 minute). The supernatant was collected. Disulfide bonds were allowed to form throughout a 1-hour incubation at 23°C. Samples were boiled before analysis using reducing (samples were treated with β-mercaptoethanol) and non-reducing (samples were not treated with β-mercaptoethanol) SDS-PAGE.

### Semi-native polyacrylamide gel electrophoresis

Samples containing 5 μM of SAVSBPM32F tetramer and various concentrations of BLA-L-SBP(A18C) (0–150 μM) were prepared. Semi-native polyacrylamide gel electrophoresis (s-native PAGE) was then performed using the following methods. The resolving gel contained 8% acrylamide and 0.27% bis acrylamide. SDS was not included in the resolving gel, stacking gel and loading buffer. 0.1% (w/v) SDS was included in the running buffer. Samples were loaded without heat and reduction treatments. Electrophoresis was performed at 4°C for 1 hour under a constant current of 30 mA.

### Modified two-dimensional polyacrylamide gel electrophoresis

The separation of protein complexes and their components in distinct dimensions was done using a modified two-dimensional polyacrylamide gel electrophoresis (M2D-PAGE). This procedure was done in two steps. A sample containing 5 μM tetrameric SAVSBPM32F and 15 μM BLA-L-SBP(A18C) was first reduced with 8 mM (final concentration) immobilized TCEP. After removal of the immobilized TCEP, the sample was allowed to form disulfide bonds and be separated using s-native PAGE. Following electrophoresis, resolved proteins were reduced within the gel by removing the gel from the gel cassette and incubating the gel in 50 ml running buffer containing 10% β-mercaptoethanol for 10 minutes at 23°C with slight agitation. The gel was first rinsed with water and then with running buffer for 1 minute at 23°C with slight agitation. Following the reduction and wash steps, the gel was inserted back into the gel cassette at a 90° angle compared to the first dimension and electrophoresed for 30 minutes at 4°C at a constant current of 35 mA. Subsequent staining, destaining and digitalization of the resulting gel were then performed.

### Kinetics measurement using the bio-layer interferometry (BLI) based BLItz system

Each binding kinetic experiment [23] was performed using the Amine Reactive 2nd Generation (AR2G) biosensors with the BLItz system (ForteBio, USA). The advanced kinetics module in the BLITz Pro software (version 1.1.0.25) was used for programming experimental steps and data acquisition. For studying the interactions between streptavidin muteins (SAVSBPM18 and SAVSBPM32) and their interacting partners, purified BLA-L-SBP(A18C), BLA-L-SBP and biotinylated BSA (BioVision, USA) were immobilized individually to separate AR2G biosensors using the amine coupling method as specified by the manufacturer. Binding of SAVSBPM32 to the immobilized ligands on the biosensors was performed under the following conditions. The initial baseline for binding was measured by placing the biosensor in Buffer A [PBS containing 0.005% Surfactant P20 (GE Healthcare) and 2 mM TCEP] for 60 seconds. Association measurements for SAVSBPM32 were obtained by placing the biosensor in solutions of purified SAVSBPM32 at different concentrations (157, 117.8, 78.5, 39.25 and 0 nM) in Buffer A and measuring the increase in the BLI signal for 400 seconds. Dissociation measurements were obtained by transferring the biosensor to a fresh solution of Buffer A and measuring the decrease in BLI signal for 800 seconds. The same procedure was then performed using SAVSBPM18. Sensorgram data were plotted on dRU/dt versus RU plot. The time constant T1 was calculated from the plot using the equation Y_max_ (1-e^(-(t-t^_0_^)/T1)^) where Y_max_ is the maximum amplitude of the BLI signal, t is the time and t_0_ is the initial time when association begins. The observed or apparent rate constant k_s_ was calculated using the equation k_s_ = (1/T1)C where C is the analyte concentration. The association rate constant k_on_ was calculated from the slope of the k_s_ versus C plot based on the equation k_s_ = k_on_C + k_off_. The dissociation rate constant k_off_ was determined using primary sensorgram data and the equation ln(R_0_/R_t_) versus t–t_0_ where R_0_ is the initial signal, R_t_ is the signal at time t, t is the time and t_0_ is the initial time. In this plot, k_off_ is equal to the slope. K_d_, the equilibrium dissociation constant, was calculated using the equation K_d_ = k_off_/k_on._ For studying the interaction between wild-type streptavidin (wtSAV) and SBP-tagged lactamase, the streptavidin (SA) biosensors were obtained from ForteBio, USA. SBP-tagged lactamase at a concentration of 158.6 nM was used in the study.

## Results

### Rationale behind the design of SAVSBPM32 and a cysteine-containing streptavidin-binding peptide tag [SBP(A18C)]

**Fig 1:**
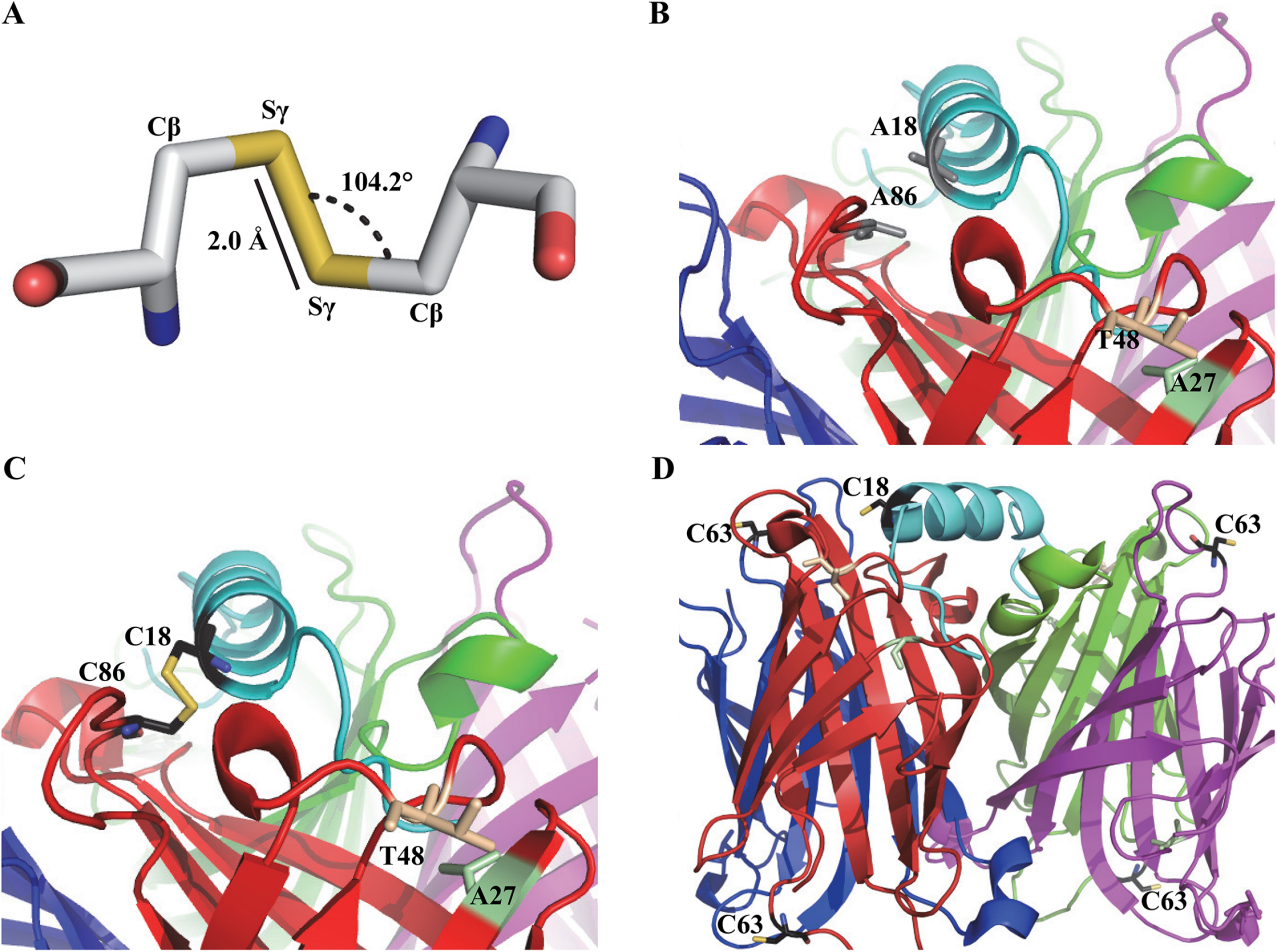
Modeled cysteine residues in streptavidin variants SAVSBPM32, SAVSBPM96 and the SBP(A18C) peptide tag. (A) Geometric and steric features of a disulfide bond formed by a pair of cysteine residues. (B) Model of the SBP-tag bound to SAVSBPM18. Residues (A86 in streptavidin and A18 in SBP) for cysteine replacement are shown in grey. Relative to wild type streptavidin, SAVSBPM18 has two mutated residues. S27A is shown in green and G48T is shown in beige. (C) Model of the disulfide-bonded SBP(A18C) and SAVSBPM32 complex with cysteine residues shown in black and the disulfide bond shown in yellow. SAVSBPM32 is generated by the introduction of the A86C mutation to SAVSBPM18. (D) Relative location of cysteine residues in the SBP(A18C)-tag: SAVSBPM96 complex. SBP and the individual streptavidin subunits are colored in cyan, red, blue, green and purple, respectively. SAVSBPM96 is a streptavidin mutein with an A63C mutation in SAVSBPM18. Models were generated using PyMOL with PDB entry 4JO6 as the starting file.

Disulfide bond formation (Fig 1, panel A) in proteins favours a Cβ-Sγ-Sγ bond angle and Sγ-Sγ bond length of 104° and 2.04 Å, respectively [24, 25]. To locate positions where cysteine replacement would satisfy these constraints, the Disulfide by Design 2 program [26] was applied to probe the recently determined crystal structure of the streptavidin:SBP-tag complex (PDB 4JO6) [15] (Table 1). This program uses an energy function to reflect the potential for the formation of disulfide bond with the lowest one having the highest possibility to form a disulfide bond. The analysis is based on a consideration of proximity and a comparison of the modelled disulfide bond geometry in comparison with idealized geometry. A86 of streptavidin and A18 of the SBP-tag were chosen for mutagenesis to cysteine residues (Fig 1, panels B and C) as they are the best candidates for three reasons. First, among all the candidates predicted, this pair has the highest probability to form a disulfide bond with the calculated energy in the range of 1.5 kcal/mol (Table 1). The second best pair (Q24 in streptavidin and G11 in SBP-tag) has a higher calculated energy of 3.67 kcal/mol. Second, to limit the loss of binding interactions caused by mutations, the residues in the SBP-tag selected for mutagenesis should not have extensive interactions with streptavidin. A18 in SBP-tag only has limited interactions with streptavidin based on the crystal structure of the streptavidin:SBP-tag complex [15]. Third, A86 in streptavidin does not play a vital role in interacting with biotin. Thus, the A86C mutation is not expected to significantly weaken the biotin-binding affinity in the mutated streptavidin. This last consideration is important because biotin needs to be an effective competitor to elute SBP-tagged proteins from the streptavidin-mutein affinity matrices after reduction of the intermolecular disulfide bond. In this study, the A86C mutation was introduced to SAVSBPM18 which binds both biotin and SBP-tag with the binding affinity in the range of 10^−8^ M [13]. The resulting mutein is named SAVSBPM32 (Table 2). The SBP(A18C) tag (Table 2) was fused to the C-terminal end of the *E*. *coli* TEM-1 β-lactamase (BLA) reporter with a 19-amino-acid linker to project the SBP(A18C) tag away from BLA. Finally, an additional Ala residue was included at the C-terminal end of the SBP-tag to enhance protein stability. The original SBP-tag ends with a proline residue, but in *E*. *coli*, proteins with a C-terminal proline residue are marked for degradation by the addition of the ssrA tag [27]. Although it is not clear whether a similar process occurs in *B*. *subtilis*, we chose to end the protein with alanine to minimize the chance for potential degradation of the tagged protein. The final construct is named BLA-L-SBP(A18C) (L stands for linker). An SAVSBPM32 variant termed SAVSBPM32F (Table 2) which included the insertion of a 9-amino-acid Flag tag (SDYKDDDDK) between E14 and A15 of SAVSBPM32 was also constructed. The serine residue (underlined) located in front of the standard Flag tag sequence serves as a spacer. Because of the introduction of this highly charged peptide sequence to SAVSBPM32, this streptavidin mutein offers better separation of streptavidin from SBP(A18C)-tagged BLA and other complexes during the analysis of the disulfide-bonded complexes using s-native PAGE and M2D-PAGE.

**Table 1:** Formation of intermolecular disulfide bonds to streptavidin-SBP complex (4JO6) as predicted by Disulfide by Design 2 (DbD2).

Streptavidin (chain: residue)	SBP tag (chain: residue)	Energy (kcal/mol)*
A: A86	Y: A18	1.49
B: A86	Z: A18	1.77
B: Q24	Z: G11	3.67
A: S45	Y: A14	5.19
B: S45	Z: E15	6.44
B: S45	Z: V14	7.77

* Calculated disulfide bond energy based on the DbD2 energy function

**Table 2:** Streptavidin muteins and sequences of the ligand tags.

**Streptavidin mutein**	**Mutations**
SAVSBPM18	S27A, G48T
SAVSBPM32	S27A, G48T, A86C
SAVSBPM32F	S27A, G48T, A86C, SDYKDDDDK insertion*
SAVSBPM96	S27A, G48T, A63C
**Ligand tag**	**Amino acid sequence** **
SBP tag	MDEKTTGWRGGHVVEG*LAGELEQLRAR*LEHHPQGQREP
SBP(A18C) tag	MDEKTTGWRGGHVVEGL**C**GELEQLRARLEHHPQGQREP**A**
PFB tag	LHHILDAQKMVWNHR
CPFB(-2) tag	LHHILD**C**QKMVWNHR

* This sequence was inserted between residues 14 and 15 of the mature SAVSBPM32 sequence.

**Bolded letters indicate the mutated residues or the extra residue added to the tag sequence. SBP sequence from L17 to R27 shown in italics represents the helical region of the SBP-tag when the tag is bound to streptavidin.

### Mutational effects on streptavidin-SBP and streptavidin-biotin interactions

It is vital to examine how the mutations in SAVSBPM32 and SBP(A18C) affect the kinetics and stability of complex formation. The binding kinetics of SAVSBPM18 and SAVSBPM32 to BLA-L-SBP and BLA-L-SBP(A18C) were determined using BLItz Biolayer Interferometry instrument [23] with BLA-L-SBP or BLA-L-SBP(A18C) muteins covalently immobilized to amine reactive second generation (AR2G) sensor. Our data indicate that the introduction of A86C mutation to SAVSBPM18 to generate SAVSBPM32 did not dramatically affect either the binding kinetics or affinity towards SBP-tag (Table 3, S1 Fig). In contrast, the A18C mutation in the SBP-tag resulted in a 9-fold reduction in binding affinity towards SAVSBPM18. The drop in affinity is mainly caused by a much higher (~10 times) rate of dissociation. However, since SAVSBPM18 has fairly high binding affinity to SBP-tag, this protein still retains strong binding affinity (K_d_ = 9.2 x 10^−8^ M) towards the SBP(A18C)-tag. As a control for the BLItz system, interaction between wild-type streptavidin and SBP was examined. Dissociation constant (4.79 x10^-9^ M, Table 3) determined by the BLItz method is comparable to the reported value (2.5 x10^-9^ M) determined by the spin-filter binding inhibition assay [12].

**Table 3:** Kinetic parameters for the interactions between streptavidin variants and the SBP ligands.

**Streptavidin**	**Binding partner**	**On-rate (M** ^**-1**^ **s** ^**-1**^ **)**	**Off-rate (s** ^**-1**^ **)**	**K**_**d**_ **(M)**
wtSAV	BLA-L-SBP	1.26 x 10^5^	6.03 x 10^−4^	4.79 x 10^−9^
SAVSBPM18	BLA-L-SBP	3.32 x 10^4^	3.41 x 10^−4^	1.03 x 10^−8^
SAVSBPM32	BLA-L-SBP	3.14 x 10^4^	3.17 x 10^−4^	1.01 x 10^−8^
SAVSBPM18	BLA-L-SBP(A18C)	3.58 x 10^4^	3.30 x 10^−3^	9.20 x 10^−8^
SAVSBPM18	Biotinylated BSA	1.01 x 10^5^	5.72 x 10^−4^	5.66 x 10^−9^
SAVSBPM32	Biotinylated BSA	4.12 x 10^4^	5.56 x 10^−4^	1.35 x 10^−8^

The A86C mutation in SAVSBPM32 ideally should not significantly affect the binding affinity between this mutein and biotin. With biotinylated BSA immobilized to the AR2G sensor for the BLItz system, the dissociation rate constant for SAVSBPM32 was very similar to that for SAVSBPM18. However, SAVSBPM32 did show a slight but significant 2.5-fold decrease in on-rate in comparison with SAVSBPM18 (Table 3, S2 Fig). The net outcome is that SAVSBPM32 still retains a fairly strong binding affinity for biotin in the order of 10^-8^M.

### Design of controls to monitor the thiol coupling process through random collisions

**Fig 2:**
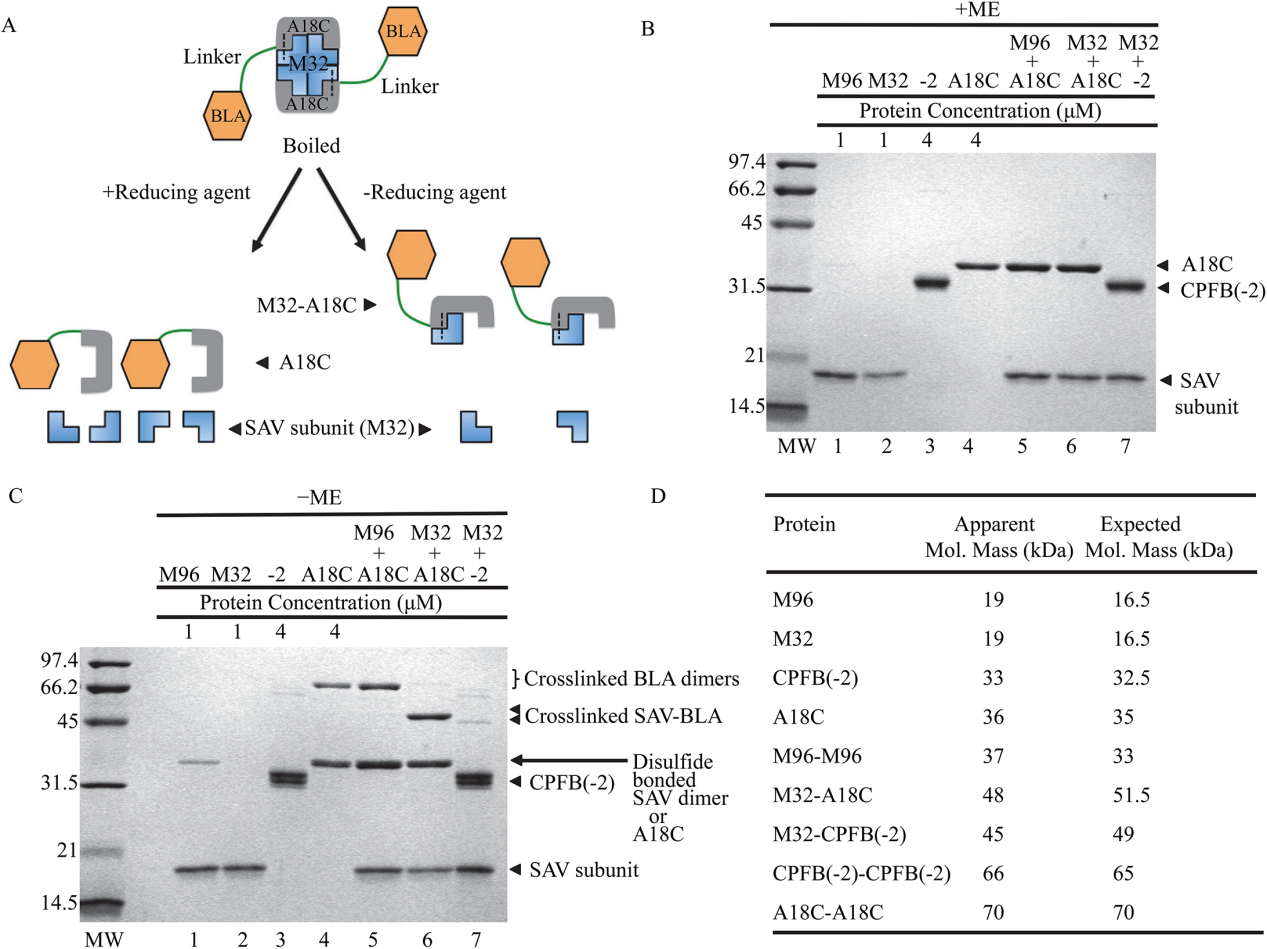
Disulfide-bond formation between SAV muteins and cysteine-containing tags. (A) Schematic of reducing and non-reducing SDS-PAGE analyses of disulfide-bonded complexes. Two BLA-L-SBP(A18C) tags (orange for BLA and grey for SBP) are covalently linked to a tetrameric SAVSBPM32 (blue) by disulfide bonds (shown as dashed lines). All samples were boiled prior to gel loading. One set of samples was analysed under reducing conditions resulting in separation of streptavidin subunits (16.5 kDa) from BLA-L-SBP(A18C) (36 kDa) as shown in panel B. The second set of samples was not reduced resulting in disulfide- bonded heterodimeric BLA-L-SBP(A18C)-SAVSBPM32 (51.5 kDa) complexes and streptavidin subunits (16.5 kDa) as shown in panel C. (B) Protein analysis by SDS-PAGE under reducing conditions. The ratio of SAV to BLA fusion proteins was 1 μM tetrameric SAV to 4 μM fusion protein in lanes 5–7. +ME indicates the presence of β-mercaptoethanol in the sample loading buffer. (C) Protein analysis by SDS-PAGE under non-reducing conditions. The ratio of SAV to BLA fusion proteins was 1 μM tetrameric SAV to 4 μM fusion protein in lanes 5–7.-ME indicates the absence of β-mercaptoethanol in the sample loading buffer. (D) Both the expected and apparent molecular masses for each species are shown. M96 and M32 represent SAVSBPM96 and SAVSBPM32, respectively. A18C and CPFB(-2) [or -2] represent BLA-L-SBP(A18C) and the non-biotinylated BLA-L-CPFB(-2), respectively.

Disulfide bond formation can occur spontaneously if two cysteine-containing proteins are present at high concentrations under an oxidizing environment. As long as the cysteine residues are surface exposed, two cysteine-containing proteins can form disulfide bonds even though these proteins do not have any binding affinity to each other. This is traditionally known as thiol coupling or thiophilic adsorption [28, 29]. In contrast, disulfide bond formation between SAVSBPM32 and BLA-L-SBP(A18C) in this study is expected to be guided or enhanced by the specific binding interactions between streptavidin and SBP. The efficiency for disulfide bond formation should be much higher than expected for random collisions between two molecules, thus making disulfide bond formation efficient even at low concentrations of SAVSBPM32 and BLA-L-SBP(A18C). In order to confirm that the formation of intermolecular disulfide bonds between the two target proteins is significantly enhanced by affinity-driven thiol coupling in comparison with thiol coupling through random intermolecular collisions, two controls were needed. For the streptavidin control, a surface exposed residue, A63, which is located far away from the SBP-tag binding pocket of SAVSBPM18 was replaced by cysteine. The resulting SAVSBPM18 derivative is termed SAVSBPM96 (Fig 1, panel D). For the peptide tag control, BLA-L-CPFB(-2) was selected [17]. This protein is a BLA fusion carrying a 15-amino-acid biotinylation tag (PFB, peptide for biotinylation) which can be enzymatically biotinylated by the *E*. *coli* biotin ligase (BirA) [19]. The CPFB(-2) tag has a surface-accessible cysteine located two residues upstream of the biotinylated lysine residue in PFB (Table 2). If this tag is not biotinylated, it has no binding affinity to streptavidin. Therefore, SAVSBPM96 in combination with BLA-L-SBP(A18C) as well as SAVSBPM32 in combination with non-biotinylated BLA-L-CPFB(-2) serve as the control reactions to monitor the degree of disulfide bond formation mediated by a thiol-coupling process through random intermolecular collisions (Fig 2, panels B and C, lanes 5 and 7).

### Formation of disulfide-bonded complexes

Both the streptavidin muteins and BLA derivatives were purified to homogeneity (Fig 2, panel B, lanes 1–4). Analysis of disulfide bond formation between streptavidin muteins and BLA derivatives was performed utilizing reducing and non-reducing SDS-PAGE. To ensure that cysteine residues in the protein samples were freely available, immobilized TCEP beads were first added to create a reducing environment. At the time for disulfide bond formation, TCEP beads were removed to create an oxidizing environment. Since each subunit in the streptavidin tetramer and each of the SBP(A18C) tag in the BLA fusions contain a cysteine, an array of potential complexes are predicted based on random collisions (Fig 2, panel A). To distinguish among these complexes, the mixtures were divided equally into two samples for analyses by SDS-PAGE in the presence or absence of β-mercaptoethanol.

In the reducing gel (Fig 2, panel B), denatured samples had only one or two protein bands representing streptavidin subunits (19 kDa), the tagged [either SBP(A18C) or CPFB(-2) tagged] BLA proteins (33–36 kDa) or a combination of these two proteins. These samples act as controls to ensure that the proteins added were free from contaminants and that each reaction contained similar amounts of protein.

In the non-reducing gel (Fig 2, panel C), denatured samples showed five predicted bands: (1) streptavidin subunits alone (19 kDa), (2) self-crosslinked streptavidin dimers (∼37 kDa), (3) streptavidin subunits covalently linked to tagged [CPFB(-2) or SBP(A18C)] BLA fusion proteins (∼46 kDa), (4) BLA-L-SBP(A18C) [or BLA-L-CPFB(-2)] monomers (~35 kDa) and (5) self-crosslinked BLA fusion protein homodimers (~70 kDa). The results show that SAVSBPM32 was able to effectively form intermolecular disulfide bonds with BLA-L-SBP(A18C) (Fig 2, panel C, lane 6, the 48-kDa crosslinked SAV-BLA protein complex). Under the same reaction conditions, only a small amount of SAVSBPM32 was observed to be covalently linked to the non-biotinylated CPFB(-2) tag (Fig 2, panel C, lane 7). These data indicate that affinity-driven thiol coupling can greatly enhance the efficiency of disulfide bond formation in comparison with random collisional processes. This conclusion is further supported by the inefficiency of disulfide bond formation between SAVSBPM96 and BLA-L-SBP(A18C) (Fig 2, panel C, lane 5). Since the cysteine residue of SAVSBPM96 is on the accessible surface and is located far away from the SBP-tag/biotin binding pocket, the absence of covalently linked heterodimers and presence of covalently linked homodimers suggests that the observed dimerization reactions are primarily the result of random collisions between the accessible cysteine residues on the surface of the two proteins. Both results indicate that effective affinity-driven thiol coupling between SAVSBPM32 and BLA-L-SBP(A18C) requires affinity between the SBP-tag and streptavidin.

It is interesting to note that under identical reaction conditions, SAVSBPM96 was observed to form disulfide-bonded homodimers but SAVSBPM32 was not (Fig 2, panel C, lanes 1 and 2). This is likely due to the difference in the position of the cysteine residue in the two variants. As previously mentioned, the cysteine residue in SAVSBPM96 is located on a surface exposed area that is outside of the SBP-tag binding pocket (Fig 1, panel D). The highly exposed location of this residue is expected to facilitate the formation of a disulfide bond between two SAVSBPM96 molecules. In contrast, self-dimerization is expected to be less efficient in SAVSBPM32 because the cysteine residue in this mutein is less accessible and the side chain is somewhat protected inside the SBP-tag binding pocket (Fig 1, panel C).

### Examination of disulfide-bonded complex formation with increasing concentration of cysteine-containing SBP-tags

**Fig 3:**
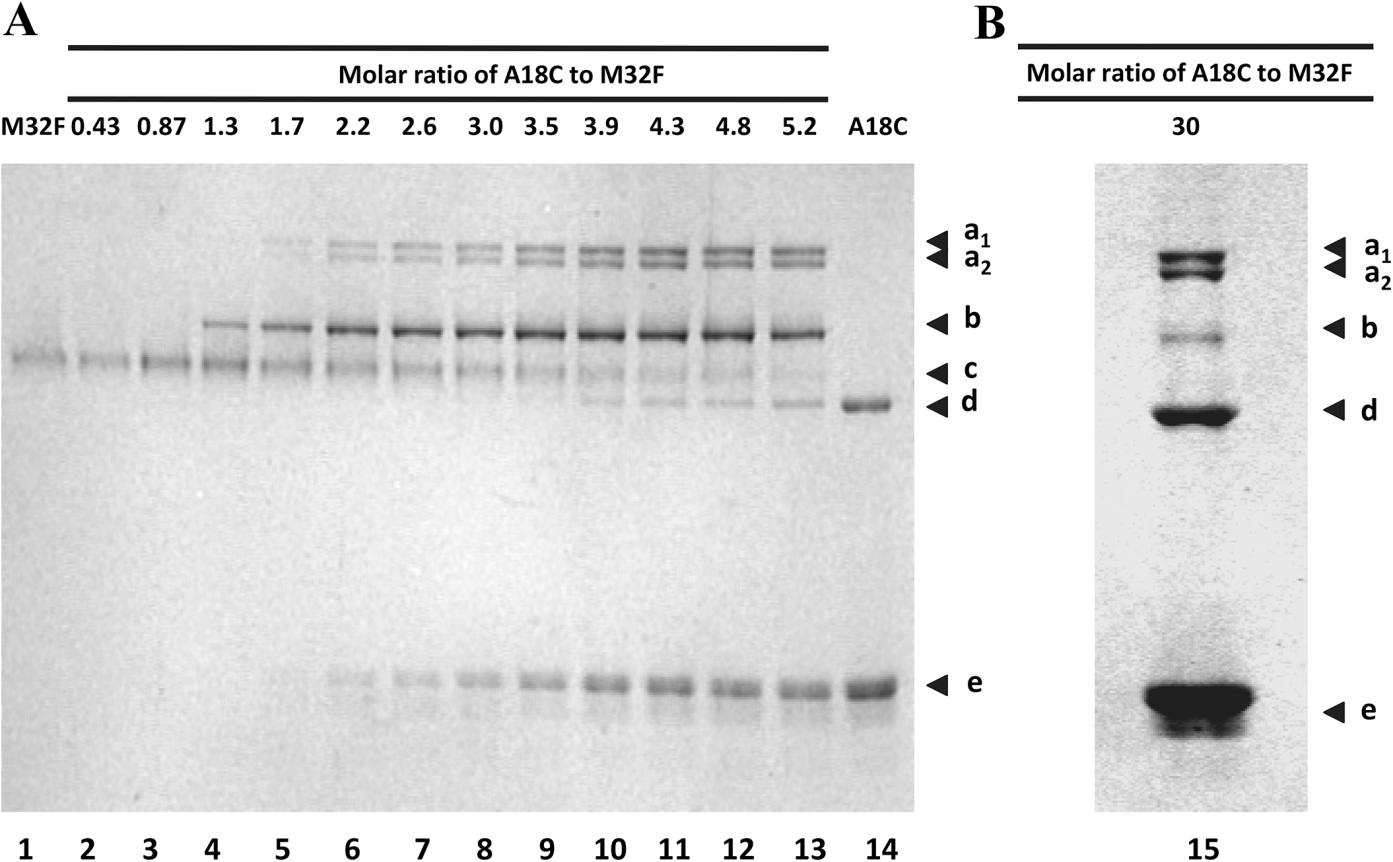
Analysis of disulfide bond formation between SAVSBPM32 and BLA-L-SBP(A18C) using semi-native polyacrylamide gel electrophoresis. Reduced BLA-L-SBP(A18C) were mixed with 5 μM reduced tetrameric SAVSBPM32F (M32F) at molar ratios ranging from 0.43:1–5.3:1 (A) and 30:1 (B). Concentrations of SAVSBPM32F and BLA-L-SBP(A18C) loaded in lanes 1 and 14 in (A) are 5 and 26.2 μM, respectively, with 12 μl samples loaded per lane. Arrowheads a1 and a2 mark the positions of the disulfide-bonded hetero-complexes a1 and a2, respectively. Arrowhead b marks the position of another disulfide-bonded hetero-complex termed complex b. Arrowheads c, d and e mark the positions of tetrameric SAVSBPM32F, homodimeric disulfide-bonded BLA-L-SBP(A18C) and the monomeric BLA-L-SBP(A18C) fusion, respectively.

A tetrameric SAVSBPM32 is expected to bind two BLA-L-SBP(A18C) molecules because a single molecule of SBP-tag binds to the biotin-binding pockets of two adjacent subunits of streptavidin [15]. SDS-PAGE under non-reducing conditions allows the demonstration of the formation of the disulfide-bonded SAVSBPM32/BLA-L-SBP(A18C) complex. However, limitations in the size resolution and absolute size accuracy of SDS-PAGE make it difficult to determine the precise stoichiometry of the two proteins in each complex. To determine the stoichiometry and composition of the complex formed, a semi-native (s-native) PAGE procedure was devised. S-native PAGE is a variation of the traditional native PAGE method by the inclusion of 0.1% SDS in the electrophoresis running buffer. The modest amount of SDS in s-native PAGE was found to be strong enough to dissociate non-covalently bound SBP-tags from SAVSBPM32 without denaturing the streptavidin tetramer (data not shown). To offer better resolution of the complexes, the FLAG-tagged (DYKDDDDK) version (SAVSBPM32F) of SAVSBPM32 was used in this study. SAVSBPM32F has a higher charge to mass ratio than SAVSBPM32. To form complexes, samples containing 5 μM of SAVSBPM32F tetramer in the presence of various concentrations (0–150 μM) of BLA-L-SBP(A18C) were prepared. The mixtures were first pretreated with immobilized TCEP beads to break any preformed disulfide bonds. The immobilized TCEP beads were then removed and the samples were left for an hour before s-native PAGE. Lanes 10–13 (Fig 3, panel A) show six distinct bands in each lane. These bands are labelled according to their increasing migration mobility through the gel as a1, a2, b, c, d, and e. Lanes 1 and 14 contain 5 μM SAVSBPM32F and 26.2 μM BLA-L-SBP(A18C) respectively. Bands c, d and e are composed of tetrameric SAVSBPM32F, disulfide bonded homodimeric BLA-L-SBP(A18C) and monomeric BLA-L-SBP(A18C), respectively. Since the concentration of SAVSBPM32F shown in lane 1 is kept constant throughout the reactions, any band shifts seen in subsequent reactions are likely not caused by the self-crosslinking of SAVSBPM32F. For BLA-L-SBP(A18C), the concentration of BLA-L-SBP(A18C) for the reaction in lane 14 is the highest concentration used throughout the titration experiment. Band shifts associated with the self-crosslinking of BLA-L-SBP(A18C) are accounted for (Fig 3, panel A, lane 14). When SAVSBPM32F and BLA-L-SBP(A18C) are mixed together, the first visible band shift (band b) occurs when the molar ratio of BLA-L-SBP(A18C) to tetrameric SAVSBPM32F is 1.3 (Fig 3, panel A, lane 4). Since this ratio is close to one SBP tag per tetramer, the composition of complex b is most likely a tetrameric SAVSBPM32F disulfide-bonded to a single BLA-L-SBP(A18C).

When the ratio of BLA-L-SBP(A18C) to SAVSBPM32F is 1.7, complex b appears to be more abundant while complexes a1 and a2 also appear (Fig 3, panel A, lane 5). This suggests that bands a1 and a2 contain protein complexes in which more than one molecule of BLA-L-SBP(A18C) is bound to a single tetramer of SAVSBPM32F. As the concentration of BLA-L-SBP(A18C) is increased up to a 4.3-fold molar excess over SAVSBPM32F, the intensities of bands a1 and a2 increase, whereas the intensity of band b only increases up to a molar ratio of 3.5 (Fig 3, panel A). The intensity of band b decreases slightly when the concentration of the SBP(A18C)-tag is increased further from 4.8 to 5.2 times the concentration of SAVSBPM32F (Fig 3, panel A, lanes 12 and 13), and the intensity drastically decreases at a ratio of 30 to 1 (Fig 3, panel B). These observations suggest that band b contains protein complexes composed of tetrameric SAVSBPM32F covalently linked to a single BLA-L-SBP(A18C) and bands a1 and a2 have more than one BLA-L-SBP(A18C) covalently linked per tetrameric SAVSBPM32F. This interpretation is consistent with the crystal structure of the SBP-tag:streptavidin complex [15] which shows that two SBP tags can bind to one streptavidin tetramer.

It is interesting to note that when BLA-L-SBP(A18C) is at a 5.2-fold molar excess over the SAVSBPM32F tetramer, band c (free form of SAVSBPM32F) is still present (Fig 3). This means that even when BLA-L-SBP(A18C) is in excess, a small portion of the SAVSBPM32F population is still unable to covalently link to the ligand. One explanation could be that some of the SAVSBPM32F tetramers are “dead” protein and unable to bind. This idea was tested by further increasing the ratio of BLA-L-SBP(A18C) to 30 times that of SAVSBPM32F. When BLA-L-SBP(A18C) is present at such high levels, almost none of the unreacted SAVABPM32F remains (Fig 3, panel B). Thus, almost all of the SAVSBPM32F tetramers are capable of binding and covalently linking to SBP(A18C)-tags.

### Stoichiometry and composition determination of the disulfide-bonded streptavidin-SBP tag complexes

**Fig 4:**
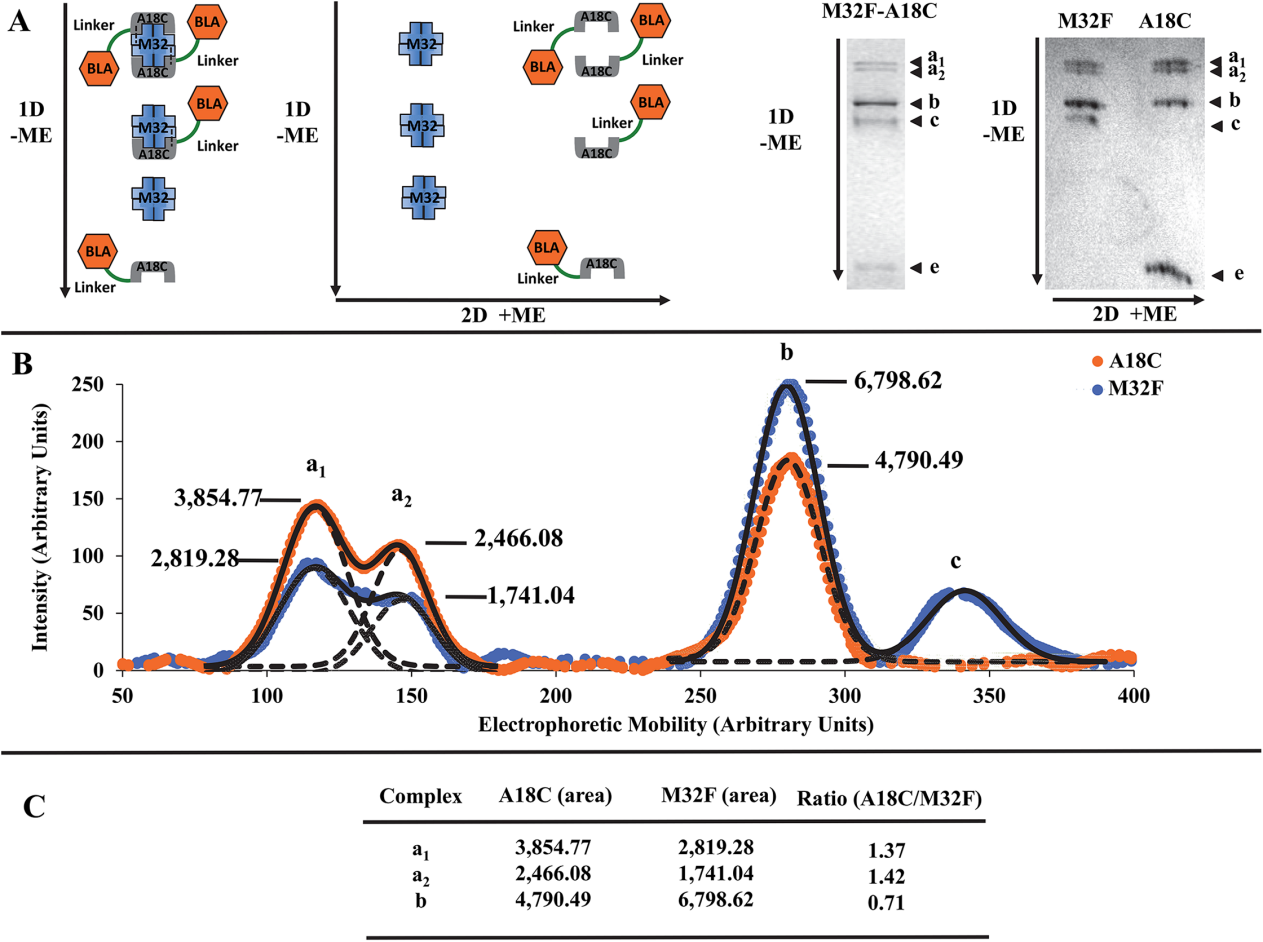
Composition determination of disulfide-bonded protein complexes by a modified two-dimensional polyacrylamide gel electrophoresis. Distinct components of the complexes formed by mixing 15 μM BLA-L-SBP(A18C) with 5 μM SAVSBPM32F were analyzed using modified two-dimensional polyacrylamide electrophoresis (M2D-PAGE). (A) Principle of the M2D-PAGE method. Protein complexes are first resolved in the first dimension using an s-native gel in the absence of reducing agent. Each of the resolved protein complexes is further separated into individual components in the second dimension in the presence of reducing agent. Order of the dimensions and the electrophoretic direction of proteins are indicated by 1D/2D and arrows, respectively. +ME and–ME indicate the presence and absence of β-mercaptoethanol, respectively. Arrowheads a1, a2, and b mark the positions of the complex a doublet and the complex b, respectively. Arrowheads c and e mark the positions of tetrameric SAVSBPM32F and monomeric BLA-L-SBP(A18C), respectively. (B) Band intensity for each component making up complexes a1, a2, b, and c was graphed. Intensities shown in orange and blue correspond to BLA-L-SBP(A18C) and SAVSBPM32F, respectively. Solid lines show the partially resolved protein peaks of the a1 and a2 complexes as a single peak. Dotted lines show the overlapping of the two partially resolved peaks of the a1 and a2 complexes. Curve fitting of the data was done using SciDAVis (http://scidavis.sourceforge.net/). (C) Relative amount of each protein in complexes a1, a2, and b is represented by the individual peak area. The BLA-L-SBP(A18C) to SAVSBPM32F ratio for a specific complex was calculated by dividing the area of the peak corresponding to BLA-L-SBP(A18C) by the area of the SAVSBPM32F peak for that particular complex.

While complex b represents protein complexes with a ratio of a single BLA-L-SBP(A18C) to a tetrameric SAVSBPM32F, the stoichiometry of the individual components in complexes a1 and a2 is unclear. To address this concern, a novel two-dimensional gel electrophoresis method was developed. In this procedure, protein complexes were separated in the first dimension by s‐native PAGE in the absence of reducing agent (Fig 4, panel A). The gel containing the resolved protein complexes was then soaked in the electrophoresis buffer containing mercaptoethanol and 0.1% SDS. The second dimension was then carried out at a 90° angle compared to the first dimension. This second step separates the individual components of the protein complexes separated under semi-native conditions during the first dimension (Fig 4, panel A). After staining the gel, the intensity of each component in the complexes was measured using ImageJ [20] and the ratio of SAVSBPM32F and BLA‐L-SBP(A18C) was determined. Results from this experiment indicate that the ratio of BLA-L‐SBP(A18C) to SAVSBPM32F is similar for the doublet (a1 = 1.37 and a2 = 1.42) and twice as large when compared to the ratio for complex b (b = 0.71) (Fig 4, panels B and C). The results not only confirm that the composition of the protein complexes a1 and a2 is the same but also provide strong evidence that each SAVSBPM32F tetramer in these complexes can bind and covalently link to two SBP(A18C)-tags.

### The SAVSBPM32 matrix works as well as the SAVSBPM18 matrix for the affinity purification of SBP-tagged proteins

**Fig 5:**
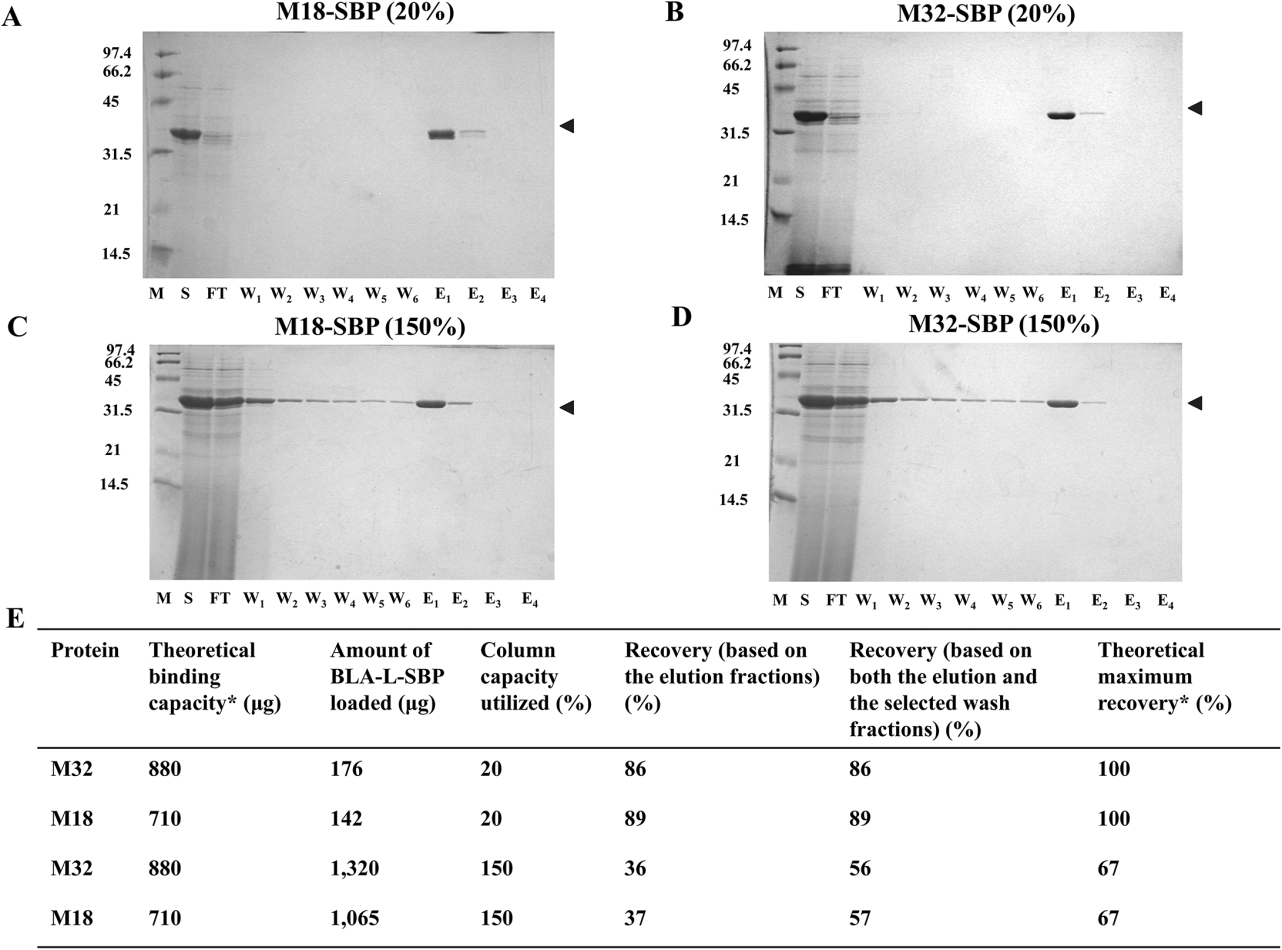
Purification of BLA-L-SBP from culture supernatant using SAVSBPM18 or SAVSBPM32 affinity matrix. Purification of BLA-L-SBP from WB800[pWB980-BLA-L-FLSBP] culture supernatant using SAVSBPM18 (panels A and C) or SAVSBPM32 (panels B and D) Affi-gel column under the non-overloaded (panels A and B) and overloaded (panels C and D) conditions, respectively. Fractions were collected and analyzed by SDS-PAGE with Coomassie blue staining. M: molecular weight markers. Numbers shown on the left represent the molecular mass (kDa) of protein markers. S: culture supernatant; FT: flow-through fraction; W1-W6: wash fractions; E1-E4: elution fractions. Arrowheads mark the position of BLA-L-SBP. Protein recoveries are shown in panel E. M18: streptavidin mutein SAVSBPM18; M32: streptavidin mutein SAVSBPM32; SBP: BLA-L-SBP. *Determination of the theoretical binding capacity and theoretical maximum percent recovery is based on the amount of BLA-L-SBP that can bind to the matrix. This value is estimated with the assumption that one BLA-L-SBP molecule binds across two subunits in either the SAVSBPM18 or SAVSBPM32 tetramer. 1 μg of SAVSBPM18 dimer (Mr = 33,037.8) or 1 μg of SAVSBPM32 dimer (Mr = 33,102) will bind 1.062 and 1.060 μg of BLA-L-SBP (Mr = 35,101.7), respectively.

An important application of SAVSBPM32 is for the affinity purification of SBP-tagged proteins. Although an affinity matrix containing SAVSBPM18 binds BLA-L-SBP effectively for affinity purification, it was previously observed that loading excess BLA-L-SBP at up to 150% of the theoretical column capacity leads to the leakage of BLA-L-SBP from the column during washing [13]. As the new SAVSBPM32 system has the capability to form disulfide bonds, it is interesting to see whether the leakage problem can be addressed. Throughout this study, leakage refers to proteins that are eluted from the column during washing. Proteins in both the flow-through and initial wash (W1) fractions are not considered as leakage because an excess amount of SBP-tagged protein was loaded, and it is not possible to distinguish protein flowing through the column and protein that is washed off the column in the first wash fraction. Purified SAVSBPM32 and SAVSBPM18 were coupled to the activated Affi-gel 15 matrices (BioRad) and used throughout the purification studies. Culture supernatants from the model proteins, BLA-L-SBP and BLA-L-SBP(A18C) were loaded onto each matrix under both overloaded and non-overloaded conditions (150% and 20% the theoretical column capacity, respectively). Samples containing BLA-L-SBP(A18C) were reduced prior to column loading. The SAVSBPM32 Affi-gel matrix showed almost identical properties as the SAVSBPM18 Affi-gel matrix in purifying SBP-tagged BLA under all conditions (Fig 5). This result is consistent with the kinetic data that SAVSBPM32 has comparable binding properties (Table 3) towards both SBP-tag and biotin as SAVSBPM18. Under the non-overloaded conditions, both columns work well with no leakage of BLA-L-SBP in both the flow-through and wash fractions (Fig 5, panels A and B).

### The SAVSBPM32 matrix works better than the SAVSBPM18 matrix in purifying SBP(A18C)-tagged BLA

**Fig 6:**
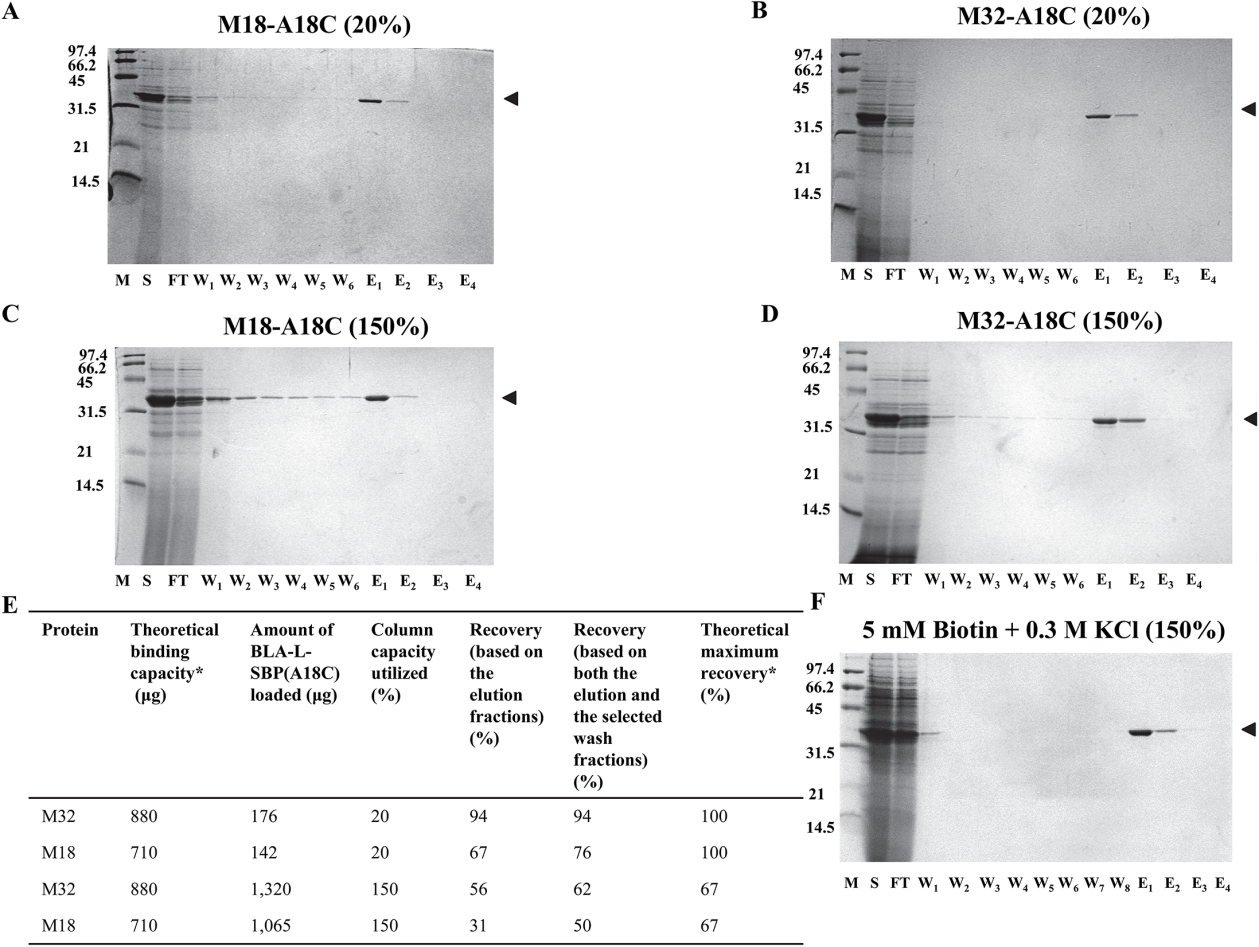
Purification of BLA-L-SBP(A18C) using SAVSBPM18 or SAVSBPM32 affinity matrix. Purification of BLA-L-SBP(A18C) from WB800[pWB980-BLA-L-SBP(A18C)] culture supernatant using SAVSBPM18 (panels A and C) or SAVSBPM32 (panels B, D and F) Affi-gel column under non-overloaded (panels A and B) and overloaded (panels C, D and F) conditions. Standard elution conditions were used with purifications shown in panels A-D. Optimized elution condition (i.e. with the addition of both 5 mM biotin and 0.3M KCl) was used as shown in panel F. Fractions were collected and analyzed by SDS-PAGE with Coomassie blue staining. M: molecular weight markers. Numbers shown on the left represent the molecular masses (kDa) of the protein markers. S: culture supernatant; FT: flow-through fraction; W1-W6: wash fractions; E1-E4: elution fractions. M18: streptavidin mutein SAVSBPM18; M32: streptavidin mutein SAVSBPM32; A18C; BLA-L-SBP(A18C). Arrowheads mark the position of BLA-L-SBP(A18C). Protein recoveries shown in panel E are prepared based on data shown in panels A-D. *Determination of the theoretical binding capacity and theoretical maximum percent recovery is based on the amount of BLA-L-SBP that can bind to the matrix. This value is estimated with the assumption that one BLA-SBP(A18C) molecule binds across two subunits in either the SAVSBPM18 or SAVSBPM32 tetramer. 1 μg of SAVSBPM18 dimer (Mr = 33,037.8) or 1 μg of SAVSBPM32 dimer (Mr = 33,102) will bind 1.063 and 1.061 μg of BLA-L-SBP(A18C) (Mr = 35,133.8), respectively.

For the purification of SBP(A18C)-tagged BLA, the SAVSBPM32 Affi-gel matrix offers much better performance than the SAVSBPM18 matrix, although both matrices can affinity purify SBP(A18C)-tagged BLA in one step to levels where no other contaminants were detectable by Coomassie blue staining (Fig 6). Even without overloading the column, leakage of SBP(A18C)-tagged BLA in the wash fractions from the SAVSBPM18 matrix was observed (Fig 6, panel A). This observation is consistent with findings that introduction of the A18C mutation to the SBP-tag reduces binding affinity by almost 9-fold with SAVSBPM18 as the binding partner (Table 3). In contrast, under identical condition using SAVSBPM32 Affi-gel matrix, no leakage of the SBP(A18C)-tagged BLA was observed in the wash fractions (Fig 6, panel B). The ability of SBP(A18C)-tagged BLA to form a disulfide bond with SAVSBPM32 in the matrix accounts for the tight retention on the column despite the reduction in noncovalent binding affinity. Under the overloaded condition, leakage of BLA-L-SBP(A18C) in the wash fractions from both matrices was observed (panels C and D). Presumably, some of the BLA-L-SBP(A18C) proteins appearing to leak from the column did not form disulfide bonds with SAVSBPM32 in the matrix. Under the typical conditions examined, the degree of leakage from the SAVSBPM32 matrix is clearly less severe than from the SAVSBPM18 matrix.

### Optimized washing conditions eliminate the leakage problem in the wash fractions

Because of the potential problems associated with the gradual leakage of protein while washing the affinity column after protein loading, it is highly desirable to identify a simple method to efficiently remove non-covalently bound proteins from the column. This is especially important if the system is to be used for the immobilization of proteins to sensor chips and other applications where the slow leakage of supposedly immobilized protein would contaminate and invalidate sensitive measurements. Purification procedures were performed under the overloaded condition. Various buffers containing different additives were used during the washing steps. Inclusion of 2% Tween 20 in the washing buffer did not improve the situation. Addition of 0.3 M KCl and 5 mM biotin individually to the washing buffer reduced the percentage of leaked BLA-L-SBP(A18C) in the wash fractions from 6% to 3% and 2%, respectively (data not shown). Inclusion of both 0.3 M KCl and 5 mM biotin in the washing buffer proved to be the most effective approach (Fig 6, panel F). Leakage was reduced to a very low level and all the non-covalently bound proteins could be removed within the first two wash fractions. After the optimized, stringent washing procedure was implemented, highly purified covalently bound BLA-L-SBP(A18C) could be eluted from the column under reducing conditions with a recovery of 47% relative to the theoretical maximum recovery of 67% (Fig 6, panel F).

### The SAVSBPM32 matrix can be applied to purify biotinylated MBP

**Fig 7:**
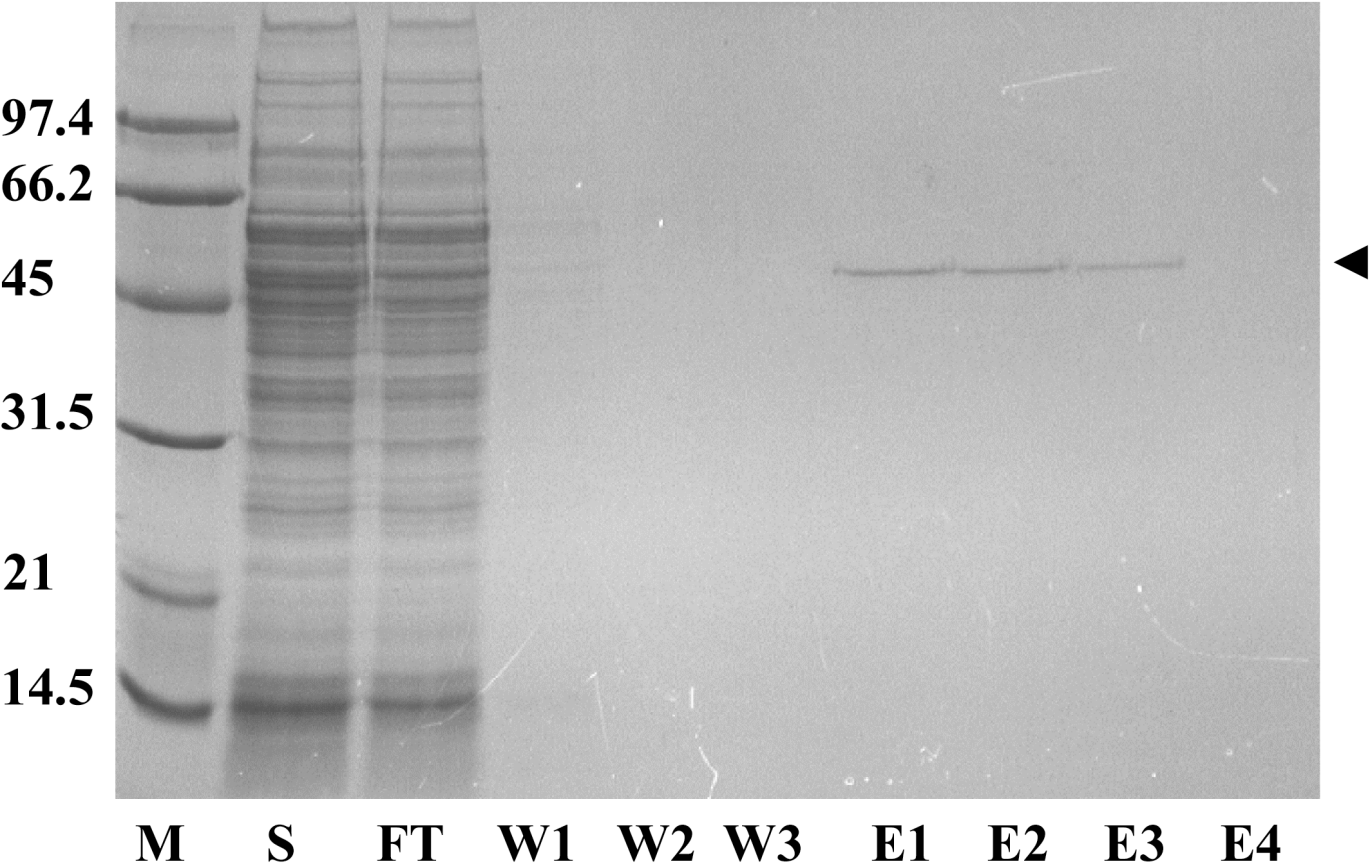
Purification of biotinylated maltose binding protein (MBP) using a SAVSBPM32 affinity column. SDS-PAGE showing purification of biotinylated MBP from a crude sample using SAVSBPM32 Affi-gel. M: molecular weight markers. S: crude sample; FT: flow-through fraction; W1-W3: wash fractions; E1-E4: elution fractions. Arrowhead marks the position of the biotinylated MBP.

Since both SAVSBPM32 and SAVSBPM18 reversibly bind biotinylated proteins (Table 3), the SAVSBPM32 matrix is expected to function in a manner similar to the SAVSBPM18 matrix for the affinity purification of biotinylated proteins. To demonstrate the utility of SAVSBPM32 for this important application, biotinylated MBP was applied to a SAVSBPM32 column at 20% of the theoretical column binding capacity. To simulate a challenging situation in purifying a low abundance target biotinylated protein from a crude protein sample, pure biotinylated MBP in small quantities was mixed with a crude extract containing large quantities of soluble cellular proteins from *B*. *subtilis*. Fig 7 shows that highly purified biotinylated MBP could be separated from the large amounts of contaminating proteins in just one step on the column. No leakage of the bound protein was observed in the wash fractions. All of the bound biotinylated MBP also appeared to be eluted, as no residual target protein was detected in the column matrix after the elution step (data not shown). The overall recovery was estimated to be over 75%. Column regeneration was achieved by thorough washing with the wash buffer.

## Discussion

### Significance and potential applications of the SAVSBPM32-SBP(A18C) tag system

Under natural conditions, interaction between streptavidin and free biotin in solution represents one of the strongest non-covalent interactions with a K_d_ of 4 x10^-14^M [8]. However, under the practical conditions, the actual interaction strength can be significantly lower. One of the possible reasons is that the biotin moieties are no longer in the free and non-conjugated form. They are commonly coupled to biomolecules or other nanostructures such as quantum dots. The same is true for streptavidin. Many studies show that the binding affinity for streptavidin-biotin at the solution-surface interface is much lower than that for the streptavidin-biotin interaction in solution. For example, the binding of biotinylated DNA to streptavidin beads has been shown to have a K_d_ value in the range of 10^−8^ to 10^-11^M [30]. The binding strength is also weakened by several orders of magnitude (in the range of 10^-10^M) when both streptavidin and biotin are immobilized to quantum dots [31]. In the presence of shear force in a flowing environment, lower binding affinity to streptavidin is observed even with free, non-conjugated biotin moieties [32]. Furthermore, high rates of dissociation for biotinylated biomolecules from streptavidin have been reported under the low pH conditions of the endosome [33]. Therefore, for many practical applications, there is a need to have stronger interactions. Traptavidin with a biotin binding affinity that is 10 times stronger than that of the natural streptavidin-biotin system can potentially fulfill some of these needs [34]. However, the traptavidin–biotin interactions are still non-covalent in nature. In this study, we demonstrate how the SAVSBPM32-SBP(A18C) system provides novel and unique capabilities to solve some of the challenges posed by many important practical applications.

At present, most engineered streptavidin systems are tailored for specific applications and are limited in their flexibility. Some systems (e.g. monomeric SAV and strep-tactin) offer reversible binding and are reusable [35–39], whereas wild-type streptavidin and traptavidin are specialized for immobilization applications [9, 34]. As a result, multiple types of streptavidin variants are required for different experiments. Working with different systems can be both time consuming and costly. With binding properties comparable to SAVSBPM18, SAVSBPM32 can be applied to purify both SBP-tagged proteins and biotinylated biomolecules (Figs 5 and 7). It can also be applied to purify or immobilize SBP(A18C)-tagged proteins (Fig 6). Users thus have the flexibility to use the same protein-coupling system for applications that require reversibility and applications that require highly stable immobilization. The SAVSBPM32-affi gel column utilized throughout this work has been used more than 20 times over a four-month period. No noticeable loss in binding has been observed. The column can be easily regenerated by a simple and gentle wash step with PBS buffer.

In addition to streptavidin-based technologies, many powerful immobilization systems including SpyTag, HaloTag, SnapTag and sortagging are also available [40–43]. The most important advantage of the SAVSBPM32 system over these other systems is that this immobilization method offers reversibility through the application of a gentle reducing agent. This feature allows the potential application of this system for the development of reusable biosensor chips, bioreactors and protein arrays. Although many purification matrices such as ion exchangers, IMAC resins and gel filtration media are reusable, it is not the case for any of the common immobilization matrices (*e*.*g*., Sortagging). Reusability makes this system a green and economically attractive technology for both purification and immobilization.

### Electrophoretic mobility difference of the streptavidin complexes with the same composition

**Fig 8:**
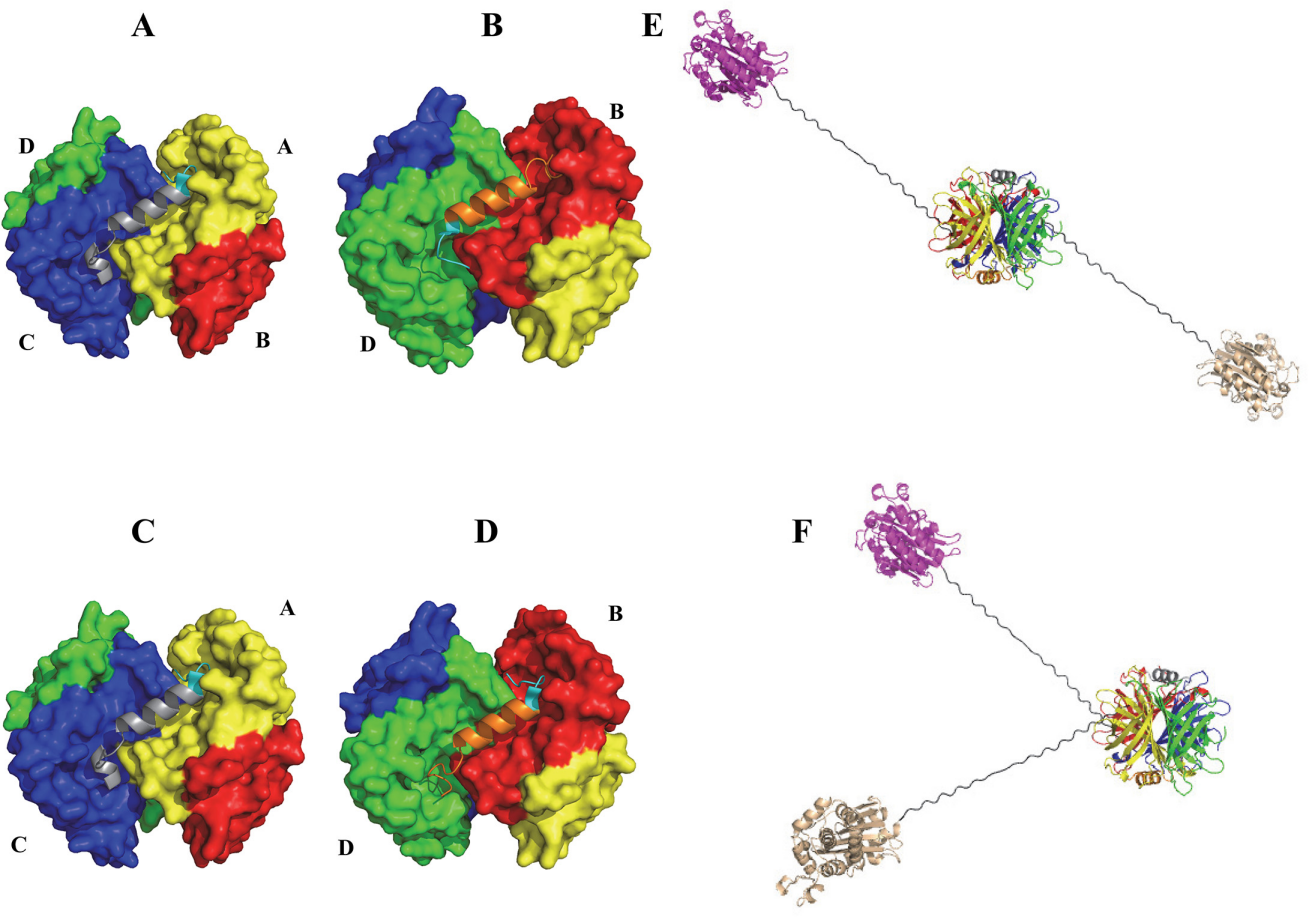
Two possible quaternary arrangements of protein complexes containing two SBP(A18C) tags per SAVSBPM32 tetramer. (A) and (B) show the orientation of the two bound SBP tags (grey and orange) in a streptavidin tetramer. The N-terminal ends of the SBP tags are shown in cyan. Binding of these tags in Arrangement 1 results in a *trans*-oriented complex (E). (C) and (D) show how SBP-tags bind to the streptavidin tetramer in Arrangement 2, resulting in a *cis*-oriented complex (F). Streptavidin is colored yellow, red, blue and green for subunits A, B, C and D, respectively. Models were generated using PyMOL with PDB entry 4JO6 as the starting file.

The novel two-dimensional polyacrylamide gel electrophoresis method (Fig 4) successfully showed that both complexes a1 and a2 contain the same composition and stoichiometry of SBP(A18C)-tagged fusion proteins and SAVSBPM32F (*i*.*e*., two molecules of BLA-L-SBP(A18C), each forming a disulfide bond with a single SAVSBPM32 tetramer). Although it is somewhat puzzling that these two complexes migrate differently on the s-native gel, differences in the quaternary structures of the two possible complexes elegantly explain the differences in their electrophoretic mobility. The crystal structure of the SBP-tag complex with wild-type streptavidin suggests that two distinct quaternary arrangements can be formed when two SBP-tags bind to a single streptavidin tetramer [15]. In Arrangement 1, which is actually observed in the crystal structure of the SBP-tag:streptavidin complex [15], the N- and C-terminal ends of a SBP-tag bind to subunits A and C of streptavidin, respectively (Fig 8, panel A), while the second SBP-tag has its N- and C-terminal ends bound to subunits D and B on the opposite face of streptavidin (Fig 8, panel B). When the two molecules of SBP-tagged fusion proteins are bound in this manner, the two BLA reporter enzymes are pointing towards opposite directions in a *trans*-configuration (Fig 8, panel E). In contrast, when Arrangement 2 occurs, the first SBP-tag has its N- and C-terminal ends arranged in the same manner as the first tag observed in Arrangement 1 (*i*.*e*., the N- and C-terminal ends bind to subunits A and C, respectively, panel C). However, the second SBP-tag in Arrangement 2 (Fig 8, panel D) binds in the opposite orientation when compared to the binding orientation of the second tag in Arrangement 1 (*i*.*e*., the N- and C-terminal ends bind to subunits B and D, respectively) (Fig 8, panel D). In this arrangement, both BLA reporter enzymes are pointing towards the same side of the tetramer in a *cis*-configuration. The *cis*- and *trans*-arrangements of the BLA reporter enzymes result in complexes with large differences in hydrodynamic radius that likely account for the observed differences in electrophoretic mobility. A similar explanation for differences in electrophoretic mobility is well characterized with respect to DNA molecules where bound proteins or unique sequences can induce bent structures [44]. This explanation predicts the presence of a doublet only when two tagged proteins are bound to one streptavidin tetramer. In contrast, a singlet should be observed when a single tagged protein is associated with a streptavidin tetramer. The observed binding profile shown in the titration study (Fig 3) is indeed consistent with these predictions.

### Efficiency of capturing two SBP(A18C) tags per tetrameric SAVSBPM32

Formation of disulfide-bonded SBP(A18C) complexes with tetrameric SAVSBPM32 facilitates the determination of the efficiency of forming the SAVSBPM32 complex with two SBP(A18C) tags. The titration study (Fig 3) indicates that high levels of SBP(A18C)-tags are needed to drive the formation of the SAVSBPM32 complexes with two SBP(A18C)-tags. When the tag to streptavidin ratio is 30:1, disappearance of the free form of the SAVSBPM32 tetramers and the formation of the majority of the complexes with two SBP(A18C)-tags per streptavidin tetramer indicate that the inefficiency of complex formation at lower tag to streptavidin ratios is not caused by the presence of inactive streptavidin tetramers (either in the misfolded state or with biotin in the biotin binding pocket) in the binding reaction. Although it is not clear why such a high tag to streptavidin ratio (30:1) is required to drive most of SAVSBPM32 into complexes with two SBP(A18C) tags bound per streptavidin tetramer, this requirement has also been shown in related studies. In a study to create tetramerized single-chain antibody (SCA) complexes using streptavidin as the tetramerization agent, mixing biotinylated single-chain antibodies with streptavidin in a 8:1 ratio only leads to a population of complexes where the majority of complexes have three single-chain antibodies per streptavidin [45]. When using biotinylated DNA as a binding ligand, the majority of streptavidin complexes have two to three DNA fragments per streptavidin even when the DNA:streptavidin ratio is five [46]. With the recent development of *cis*- and *trans*-divalent streptavidin [47, 48], steric hindrance is shown to be a factor accounting for the observed negative cooperativity for binding two biotinylated molecules to the *cis*-sites in streptavidin. In the SAVSBPM32-SBP(A18C) system described above, each bound SBP-tag occupies the two biotin binding pockets located on the same side of streptavidin (Fig 8). As a result, each SBP-tag appears to bind independently of the other and so steric hindrance is not expected to be a major factor accounting for the observed lower binding efficiency. The predicted structure of the SBP(A18C)-tag suggests another explanation for the importance of a high ligand to streptavidin ratio to drive complex formation. Kinetics measurements indicate that the introduction of the A18C mutation into the SBP-tag increases the rate of dissociation from SAVSBPM18 by almost 10 times. Since the A86C mutation in SAVSBPM32 does not change the binding properties towards the SBP-tag, it is expected that SAVSBPM32 would behave like SAVSBPM18 and bind the SBP(A18C)-tag with lower binding affinity. Although the A18C mutation in the SBP-Tag was designed to be minimally disruptive to binding interactions with SAVSBPM18 and SAVSBPM32, the replacement of A18 with cysteine may disrupt the helical secondary structure observed in the central portion (residues L17-R27) of the SBP-Tag bound to streptavidin (S3 Fig, panel A). Prediction of the secondary structure of SBP(A18C) (S3 Fig, panel B) by the PEP-FOLD program [49] suggests that the replacement of A18 by cysteine may increase the tendency of the tag to form a kink around the cysteine residue (S3 Fig, panel C). Cysteine has a much lower propensity to be in a helical structure than alanine [50, 51]. Furthermore, glycine, a strong helix breaker residue [50, 51], was present at position 19 of SBP-tag (Table 2). The combination of these two residues (Cys-Gly) may increase the chance of this region to adopt a kink at the beginning of the helical structure. In this conformation, the cysteine residue may not position properly to form the expected disulfide bond. Consequently, the N-terminal portion of the SBP tag may not optimally position itself to the binding pocket, thus leading to the observed increase in off-rate. Structure determination of the complex formed between SAVSBPM32 and SBP(A18C) is currently under way and may shed further light on the conformation of this critical region of SBP(A18C).

This explanation is consistent with the chromatographic behavior of SBP-tagged BLA on the SAVSBPM32 column. Based on the observed wash and elution profiles, the binding of SBP(A18C)-tags to SAVSBPM32 can be divided into two different binding modes. The first one is a high-affinity binding mode in which the SBP(A18C)-tag binds to streptavidin as observed in the crystal structure of the streptavidin-SBP tag complex and favors the formation of disulfide-bonded complexes. The second, lower affinity binding mode is characterized by the formation of a kink near the beginning of the helical region of the SBP(A18C)-tag. When bound in this manner, an intermolecular disulfide bond does not form efficiently and the non-covalently bound proteins can be eluted off from the column by the inclusion of 5 mM biotin and 0.3 M KCl in the wash buffer in the absence of reducing agents. SBP(A18C)-tags bound in this mode will occupy the biotin binding pocket and will prevent the binding of another SBP(A18C)-tag to the same site. Therefore, under the binding conditions with the SBP(A18C)-tag in excess, each SAVSBPM32 tetramer in solution may actually be saturated with two SBP(A18C)-tags although not all the bound SBP(A18C) tags can form the disulfide-bonded structure. The apparent lower binding efficiency observed in the titration study (Fig 3) reflects that tags bound to streptavidin in the lower affinity binding mode are stripped off from the complexes because of the inclusion of 0.1% SDS in the electrophoretic buffer in the semi-native gel system.

## Supporting Information

S1 Fig Determination of the kinetic parameters (on-rate and off-rate) of the interaction between streptavidin (or its muteins) and SBP [or SBP(A18C)] tagged β-lactamase. For studying interactions of SAVSBPM32 and SAVSBPM18 with SBP-tagged BLA and its derivative, BLA-L-SBP and BLA-SBP(A18C) were immobilized to biosensor chips. SAVSBPM32 and SAVSBPM18 functioned as analytes. For studying interaction between wtSAV and BLA-L-SBP, the streptavidin (SA) biosensor was used and BLA-L-SBP functioned as an analyte. (A) Linearized data from sensorgrams for the determination of the on-rate (slope of the plot). (B) Linearized data from sensorgrams for the determination of off-rate (slope of the plot). Data plotted for M18:A18C and M32:SBP in (B) are the average of three replicates ± SEM. Data plotted for wtSAV:SBP and M18:SBP are from one trial (B). wtSAV: wild-type streptavidin; M18: streptavidin mutein SAVSBPM18; M32: streptavidin mutein SAVSBPM32; SBP: BLA-L-SBP (β-lactamase tagged with SBP tag); A18C: BLA-L-SBP(A18C) (β-lactamase tagged with SBP tag).
(DOCX)

S2 Fig Determination of the kinetic parameters (on-rate and off-rate) of the interaction between streptavidin muteins and biotinylated BSA. Biotinylated BSA was immobilized to biosensor chips and streptavidin muteins (SAVSBPM18 or SAVSBPM32) functioned as analytes. (A) Linearized data from sensorgrams for the determination of the on-rate (slope of the plot). (B) Linearized data from sensorgrams for the determination of off-rate (slope of the plot). Data plotted in (B) are the average of three replicates ± SEM. M18: streptavidin mutein SAVSBPM18; M32: streptavidin mutein SAVSBPM32.
(DOCX)

S3 Fig Models of SBP- and SBP(A18C)-tags. (A) The three-dimensional structure of the SBP-tag showing the helical region (L17—R27) which functions as a spacer to position the N- and C-terminal peptides to the streptavidin-binding pocket. This structure was prepared based on the 4JO6 PDB file. (B) One of the modeled structures of SBP(A18C)-tag generated from the PEP-FOLD webserver. The tag has a helical region similar to the one observed in the wild type SBP-tag. (C) Another model of the SBP(A18C)-tag generated from the PEP-FOLD webserver. In this modeled structure, a kink is introduced resulting in a shorter helical region. A18 in SBP and C18 in SBP(A18C) are shown in space-filling representation.
(DOCX)
