# Supplementary material for: Engineering Streptavidin and a Streptavidin-Binding Peptide with Infinite Binding Affinity and Reversible Binding Capability: Purification of a Tagged Recombinant Protein to High Purity via Affinity-Driven Thiol Coupling
Source: PLoS One. 2015 Sep 25;10(9):e0139137. doi: 10.1371/journal.pone.0139137 (PMC4583386; doi:10.1371/journal.pone.0139137)
Supplement: S1 Fig — For studying interactions of SAVSBPM32 and SAVSBPM18 with SBP-tagged BLA and its derivative, BLA-L-SBP and BLA-SBP(A18C) were immobilized to biosensor chips. SAVSBPM32 and SAVSBPM18 functioned as analytes. For studying interaction between wtSAV and BLA-L-SBP, the streptavidin (SA) biosensor was used and BLA-L-SBP functioned as an analyte. (A) Linearized data from sensorgrams for the determination of the on-rate (slope of the plot). (B) Linearized data from sensorgrams for the determination of off-rate (slope of the plot). Data plotted for M18:A18C and M32:SBP in (B) are the average of three replicates ± SEM. Data plotted for wtSAV:SBP and M18:SBP are from one trial (B). wtSAV: wild-type streptavidin; M18: streptavidin mutein SAVSBPM18; M32: streptavidin mutein SAVSBPM32; SBP: BLA-L-SBP (β-lactamase tagged with SBP tag); A18C: BLA-L-SBP(A18C) (β-lactamase tagged with SBP tag). (DOCX) [file pone.0139137.s001.docx]

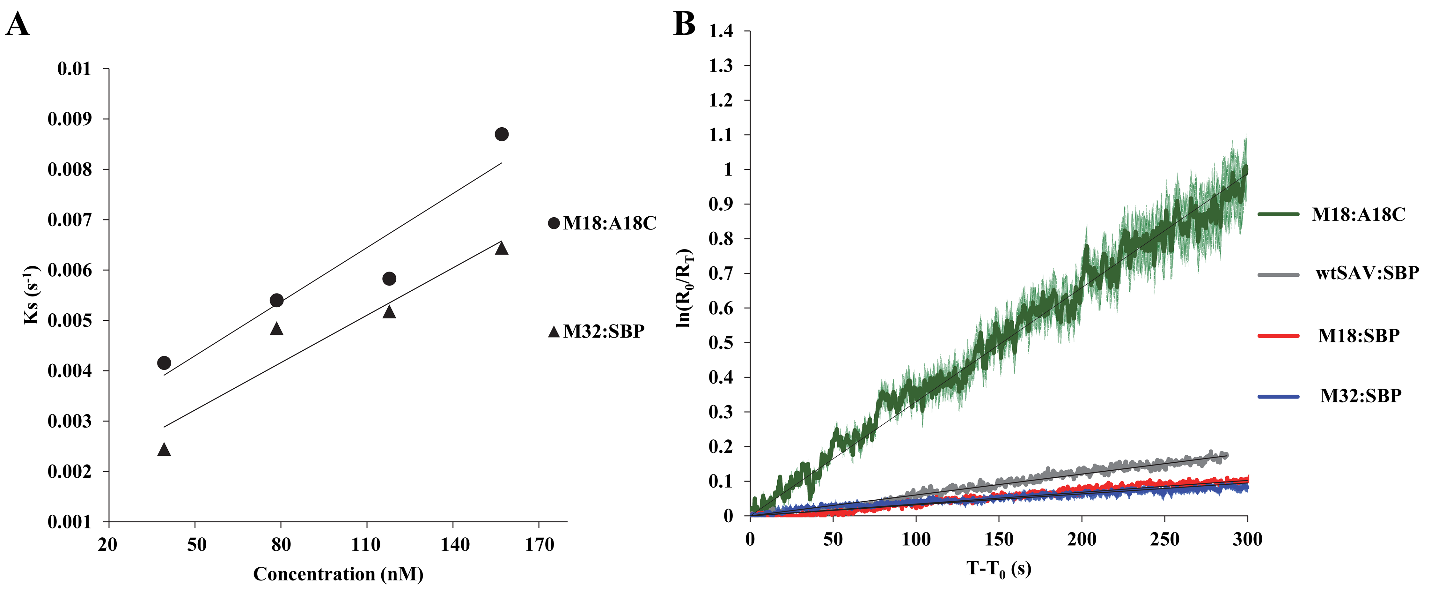


**S1 Fig. Determination of the kinetic parameters (on-rate and off-rate) of the interaction between streptavidin (or its muteins) and SBP [or SBP(A18C)] tagged β-lactamase.**

BLA-L-SBP and BLA-SBP(A18C) were immobilized to biosensor chips. Wild-type streptavidin, SAVSBPM32 and SAVSBPM18 functioned as analytes. (A) Linearized data from sensorgrams for the determination of the on-rate (slope of the plot). (B) Linearized data from sensorgrams for the determination of off-rate (slope of the plot). Data plotted for M18:A18C and M32:SBP in (B) are the average of three replicates ± SEM. Data plotted for wtSAV:SBP and M18:SBP are from one trial (B). wtSAV: wild-type streptavidin; M18: streptavidin mutein SAVSBPM18; M32: streptavidin mutein SAVSBPM32; SBP: BLA-L-SBP (β-lactamase tagged with SBP tag); A18C: BLA-L-SBP(A18C) (β-lactamase tagged with SBP tag).
