# Supplementary material for: Engineering Streptavidin and a Streptavidin-Binding Peptide with Infinite Binding Affinity and Reversible Binding Capability: Purification of a Tagged Recombinant Protein to High Purity via Affinity-Driven Thiol Coupling
Source: PLoS One. 2015 Sep 25;10(9):e0139137. doi: 10.1371/journal.pone.0139137 (PMC4583386; doi:10.1371/journal.pone.0139137)
Supplement: S2 Fig — Biotinylated BSA was immobilized to biosensor chips and streptavidin muteins (SAVSBPM18 or SAVSBPM32) functioned as analytes. (A) Linearized data from sensorgrams for the determination of the on-rate (slope of the plot). (B) Linearized data from sensorgrams for the determination of off-rate (slope of the plot). Data plotted in (B) are the average of three replicates ± SEM. M18: streptavidin mutein SAVSBPM18; M32: streptavidin mutein SAVSBPM32. (DOCX) [file pone.0139137.s002.docx]

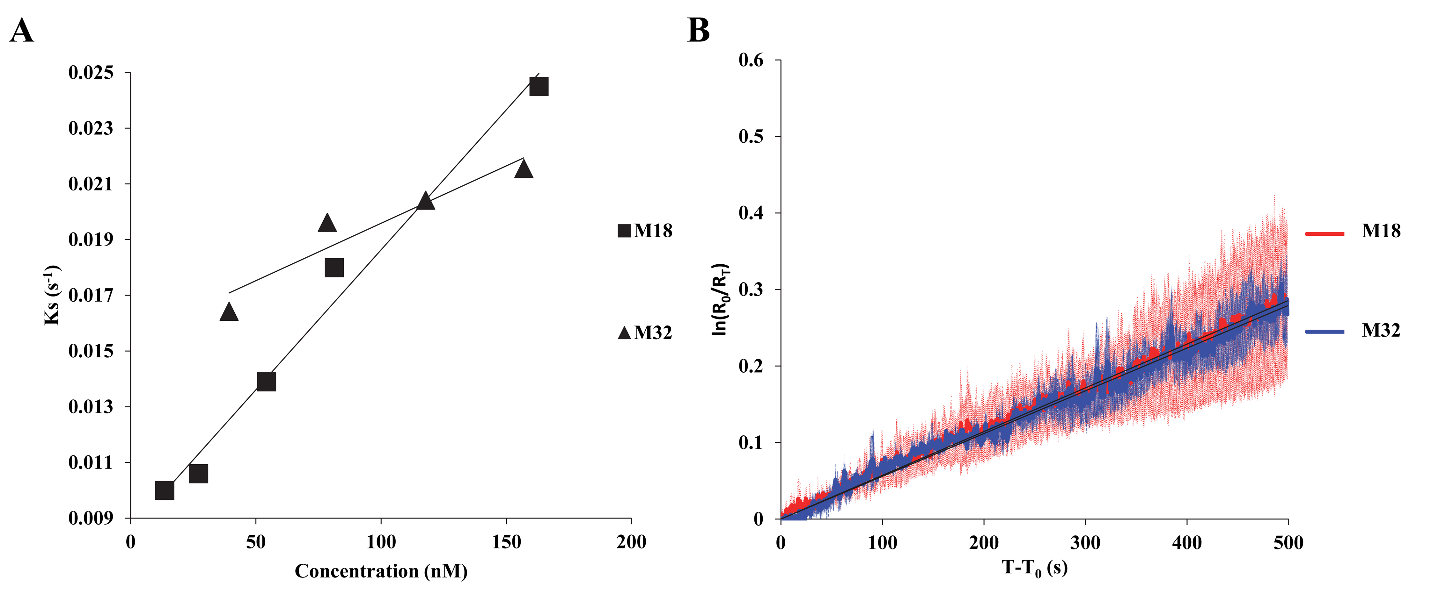


**S2 Fig. Determination of the kinetic parameters (on-rate and off-rate) of the interaction between streptavidin muteins and biotinylated BSA**

Biotinylated BSA was immobilized to biosensor chips and streptavidin muteins (SAVSBPM18 or SAVSBPM32) functioned as analytes. (A) Linearized data from sensorgrams for the determination of the on-rate (slope of the plot).  (B) Linearized data from sensorgrams for the determination of off-rate (slope of the plot). Data plotted in (B) are the average of three replicates ± SEM. M18: streptavidin mutein SAVSBPM18; M32: streptavidin mutein SAVSBPM32.
