# Supplementary material for: Engineering Streptavidin and a Streptavidin-Binding Peptide with Infinite Binding Affinity and Reversible Binding Capability: Purification of a Tagged Recombinant Protein to High Purity via Affinity-Driven Thiol Coupling
Source: PLoS One. 2015 Sep 25;10(9):e0139137. doi: 10.1371/journal.pone.0139137 (PMC4583386; doi:10.1371/journal.pone.0139137)
Supplement: S3 Fig — (A) The three-dimensional structure of the SBP-tag showing the helical region (L17—R27) which functions as a spacer to position the N- and C-terminal peptides to the streptavidin-binding pocket. This structure was prepared based on the 4JO6 PDB file. (B) One of the modeled structures of SBP(A18C)-tag generated from the PEP-FOLD webserver. The tag has a helical region similar to the one observed in the wild type SBP-tag. (C) Another model of the SBP(A18C)-tag generated from the PEP-FOLD webserver. In this modeled structure, a kink is introduced resulting in a shorter helical region. A18 in SBP and C18 in SBP(A18C) are shown in space-filling representation. (DOCX) [file pone.0139137.s003.docx]

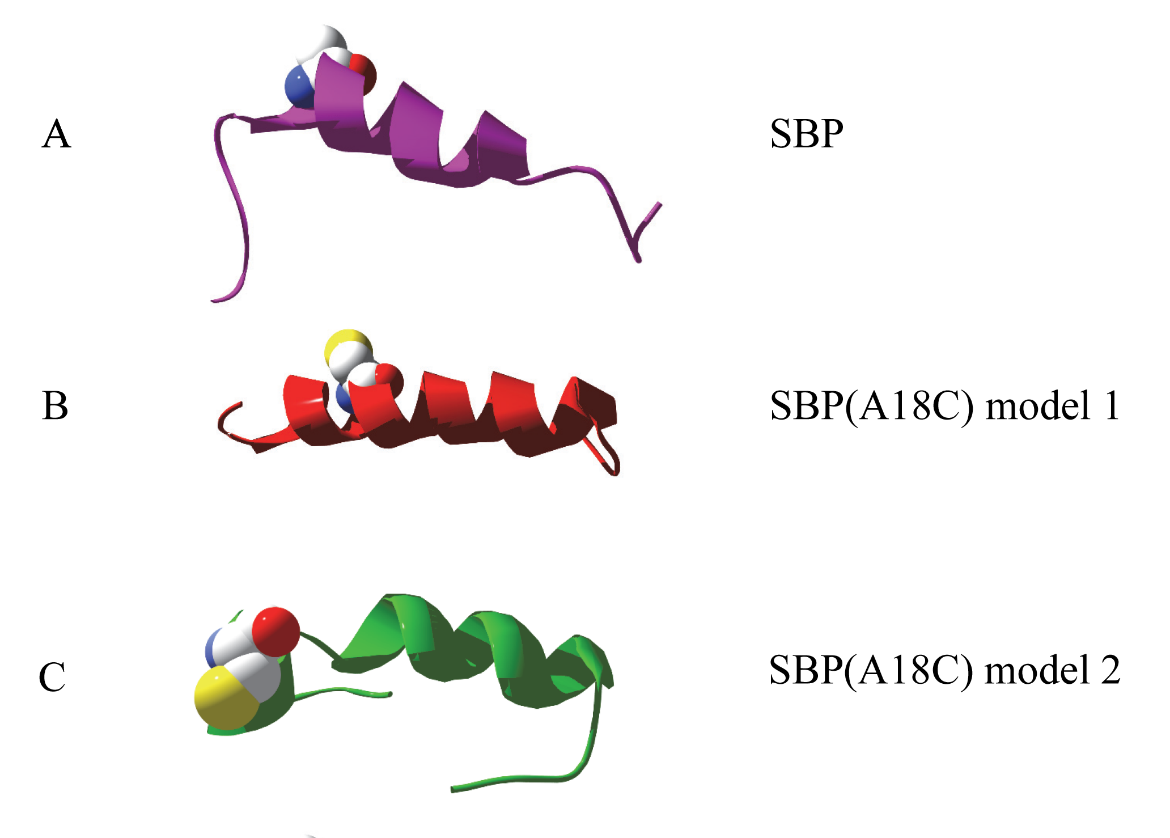


**S3 Fig. Models of SBP- and SBP(A18C)-tags.**

(A) The three-dimensional structure of the SBP-tag showing the helical region (L17 - R27) which functions as a spacer to position the N- and C-terminal peptides to the streptavidin-binding pocket. This structure was prepared based on the 4JO6 PDB file. (B) One of the modeled structures of SBP(A18C)-tag generated from the PEP-FOLD webserver. The tag has a helical region similar to the one observed in the wild type SBP-tag. (C) Another model of the SBP(A18C)-tag generated from the PEP-FOLD webserver. In this modeled structure, a kink is introduced resulting in a shorter helical region. A18 in SBP and C18 in SBP(A18C) are shown in space-filling representation.
